# Parent‐Reported Relations Between Vocabulary and Motor Development in Infancy: Differences Between Verbs and Nouns

**DOI:** 10.1111/infa.12638

**Published:** 2024-11-21

**Authors:** Kelsey L. Frewin, Sarah A. Gerson, Ross E. Vanderwert, Chiara Gambi

**Affiliations:** ^1^ School of Psychology University of East Anglia Norwich UK; ^2^ School of Psychology Cardiff University Centre for Human Developmental Science, Cardiff University Cardiff UK; ^3^ School of Psychology Cardiff University Cardiff UK; ^4^ Department of Psychology University of Warwick Coventry UK

**Keywords:** infancy, motor development, noun learning, verb learning, vocabulary

## Abstract

During early development, increases in vocabulary are related to gains in motor ability, above and beyond the effects of maturation alone. However, little is known about the association between motor development and children's early acquisition of different types of words. We examined whether motor development is differentially associated with concurrent verb and noun vocabulary in 83 infants aged 6‐ to 24‐months‐old. We asked caregivers to complete parent‐report measures of vocabulary acquisition and motor development. Analyses revealed that the association between word comprehension and motor development significantly differed for verb and nouns. Infants' verb comprehension was more strongly associated with motor development than noun comprehension. We discuss how infants' own motor actions may provide cues that are especially important for narrowing down the meaning of novel verbs.

## 1 Introduction

During language development, children find verbs more challenging to learn than nouns in most languages, with verbs appearing later and less frequently in children's vocabularies (Gentner 1982, 2006; Gleitman and Gleitman 1992; Tomasello 1992a). This finding is not universal, with children learning “verb‐friendly” languages (e.g., where nouns can be dropped and verbs are often used in isolation) reported to learn nouns and verbs at similar rates and frequencies (Choi and Gopnik 1995; Tardif 1996; Tardif, Gelman, and Xu 1999). Nevertheless, the noun bias has been reported in children speaking English (the language examined in this study), German, Kaluli, Japanese, Mandarin, and Turkish (Gentner 1982) and in Spanish, Italian, French, Dutch, Hebrew, and Korean (Bornstein et al. 2004; Caselli et al. 1995; Jackson‐Maldonado et al. 1993; Rescorla et al. 2013). This noun bias is often explained by the types of concepts that early verbs and nouns tend to represent. Nouns refer to concrete objects and entities that are effortlessly individuated whereas verbs denote abstract concepts that can only be perceived for brief periods of time (Gentner and Boroditsky 2001; Gleitman 1990; Gleitman and Gleitman 1992; Snedeker and Gleitman 2004; Tomasello 1995).^1^ Nonetheless, several verbs appear early in children's word production and by 2 years of age, children know the meanings of many verbs (Fenson et al. 1994; Goldin‐Meadow, Seligman, and Gelman 1976; Mani and Huettig 2012), even when they are exposed to noun‐friendly languages like English and German. So, how do infants identify verb referents and learn their meanings? One possible source of information may be infants' own motor actions.

As infants acquire new motor abilities, they learn to perform novel actions that elicit verb labelling from caregivers (Tamis‐LeMonda et al. 2019; West et al. 2022, 2023), generating unique opportunities for verb learning. Influenced by embodied accounts of language acquisition, dynamic systems theory, and developmental cascades perspectives (Iverson 2010, 2021, 2022; Oakes and Rakison 2020; Piaget 1952; Thelen 2000a, 2000b; Thelen and Smith 1996), studies have explored whether language learning may be supported by gains in infants' motor abilities. Within these view points, infants' language learning is embodied—that is, developing sensorimotor abilities affect how children process incoming information and engage with the world around them, thus influencing language development. These perspectives assume that learning, both in the language and motor (and other cognitive) domains, is non‐linear and emerges in context in response to a series of infant–environment interactions. In contrast, these perspectives tend to downplay the role of neural maturation and the idea that development can be described as a linear sequence of milestones which are “pre‐set” to emerge at particular ages. According to embodied accounts, instead, infants' varied interactions likely explain large inter‐individual variability in motor and language development. For example, the process of word learning appears to be initially slow and gradual, but later proceeds in spurts, as shown with both lab‐based comprehension tasks (Bergelson 2020) and parent‐reported word production data (Goldfield and Reznick 1990).

In exploring ties between language and motor development, many previous studies have focused on investigating correlations between infants' motor abilities and general language outcomes (e.g., vocabulary size; Gonzalez, Alvarez, and Nelson 2019). We advance this exploration by cross‐sectionally investigating whether infants' motor development (as reported by their parents) is more highly correlated with their verb comprehension than noun comprehension, in infants aged 6‐ to 24‐month.

## 2 Links Between Advances in Motor Skills and Language Development

Greater motor ability is often associated with larger vocabularies, concurrently and longitudinally (see Gonzalez, Alvarez, and Nelson 2019, for a review). Often, these findings are reported in relation to gross and fine motor abilities. Gross motor skills refer to large limb actions involving the arms, legs, and torso; fine motor skills require precise hand coordination (e.g., in grasping actions) but are considered distinct from intentionally communicative gestures (Alcock and Krawczyk 2010). Parent‐reported measures of gross motor skills have been found to correlate positively with the number of words infants understand and say at 21‐months‐old (Alcock and Krawczyk 2010; Valla et al. 2020). The emergence of independent sitting and self‐locomotion are thought to effect meaningful changes in infants' linguistic and communicative development. For example, unaided sitting improves infants' visual access to their surroundings and ability to explore objects, resulting in increased caregiver interactions (Franchak, Kretch, and Adolph 2018; Kretch, Franchak, and Adolph 2014; Rochat and Goubet 1995; Soska and Adolph 2014). The cascading impact of these changes are reflected in language development, with infants that learn to sit unaided earlier in infancy possessing larger productive vocabularies as they approach toddlerhood (Oudgenoeg‐Paz, Volman, and Leseman 2012). Similarly, the transition from crawling to walking affords infants visual and hands‐free access to distal locations, objects, and people (Dosso and Boudreau 2014; Karasik, Tamis‐Lemonda, and Adolph 2011). This transition is marked by increased communication from caregivers as infants engage in more complex social bids (Clearfield 2011; Clearfield, Osborne, and Mullen 2008; Karasik, Tamis‐Lemonda, and Adolph 2014; Schneider and Iverson 2022). During this time, infants begin producing more gestures and vocalizations, which caregivers of early walkers are responsive to (West and Iverson 2021). Walking infants also understand and say more words than age‐matched crawling infants (He, Walle, and Campos 2015; Walle and Campos 2014, but see, Moore et al. 2019). Earlier acquisition of independent walking is also associated with larger productive vocabularies during early childhood (Oudgenoeg‐Paz, Volman, and Leseman 2012). In a study exploring links between walking status and vocabulary size, He, Walle, and Campos (2015) replicated Walle and Campos' (2014) findings that walking infants have larger receptive and productive vocabularies than crawling infants of the same age (in both Chinese and American infants), for both noun and non‐noun vocabulary. In contrast, Karasik, Tamis‐Lemonda, and Adolph (2014) found no differences in vocabulary sizes between aged‐matched walking and crawling infants. Some studies also report that associations between gross motor and language development reduce in strength or no longer hold when accounting for covariates such as general cognitive ability (Houwen et al. 2016) and other motor (e.g., fine, oral) and gesture skills (Alcock and Krawczyk 2010). Though other studies have found that associations hold when accounting for covariates such as infant sex, SES, and maternal education (Muluk, Bayoğlu, and Anlar 2016). In sum, there is good evidence that gross motor skills are associated with vocabulary development, but it is unclear how strong this relation is and what factors modulate it.

Advances in fine motor abilities have also been associated with larger receptive and expressive vocabularies during infancy (Alcock and Krawczyk 2010; Houwen et al. 2016). Object manipulation facilitates infants' acquisition of object knowledge and properties such as shape, texture, and weight (Rochat 1989; Ruff 1984). During labeling moments, fine motor skills enable infants to hold and manipulate objects which occupy their visual field and focus their attention on the referent (Pereira, Smith, and Yu 2014; Yu and Smith 2012). Four‐ and six‐month‐old infants who are more skilled at manipulating objects typically have also been found to have larger vocabularies later during infancy and toddlerhood (Choi et al. 2018; Ruddy and Bornstein 1982; Zuccarini et al. 2018). Houwen et al. (2016) found that associations between fine motor skills and vocabulary (receptive and productive) during early development hold even after controlling for general cognitive ability. However, Alcock and Krawczyk (2010) found that associations between fine motor skills and vocabulary size did not hold after controlling for other motor abilities (e.g., gross, oral), gesture skills, and parental employment. Thus, while generally fewer studies have looked at fine (as opposed to gross) motor skills in association with language development, there is some evidence for an association between fine motor skills and vocabulary size, but once again it is unclear what factors modulate this relation.

## 3 Verbs and Motor Behaviors

Seemingly, advances in motor ability enable infants to explore objects and environments in novel ways and caregivers respond to these changes sensitively by responding with relevant labels (Iverson 2010, 2021, 2022; Oakes and Rakison 2020; Piaget 1952; Thelen 2000a, 2000b; Thelen and Smith 1996). Below we discuss how motor skill attainments might create novel opportunities for noun and verb learning. Across interactions, children likely notice co‐occurrences between their self‐produced actions and verbs produced by their caregivers, via sensorimotor learning mechanisms (Antognini and Daum 2019; Gerson, Bekkering, and Hunnius 2015; Hunnius and Bekkering 2014) and domain‐general statistical learning mechanisms (Samuelson and Smith 1998; Smith, Jones, and Landau 1996; Smith and Yu 2008; Yu and Smith 2007). Caregivers are sensitive to their infant's actions, responding to them often, using a diverse range of verbs (West et al. 2022), and temporally and semantically aligning their utterances with infants' bodily actions (e.g., “Are you *walking*?” during a walking bout) or object manipulations (e.g., “That's a *cup*” when a cup is grasped; Nomikou, Koke, and Rohlfing 2017; Suarez‐Rivera, Linn, and Tamis‐LeMonda 2022; Tamis‐Lemonda, Kuchirko, and Tafuro 2013; Tamis‐LeMonda et al. 2019; West et al. 2022, 2023).

West and colleagues (2022), in particular, demonstrated that parents' verb input is tightly linked to the specific actions that their infant is performing in the moment. Mothers and infants were recorded at home interacting during everyday activity. All of the infants' actions around a verb labeling event (before and after) were coded; either as a whole body action if it involved a large movement of the legs (e.g., kicking, jumping), locomotion (e.g., walking), or changed posture (e.g., squatting, sitting down) or coded as a manual action if it involved use of the fingers, hands or arms (e.g., stacking, shaking, waving, clapping). Mothers' verb input was coded as a precise match if the verb exactly mapped onto the action performed (e.g., the infant kicked a ball when their mother said “kick”), coded as imprecise if infants performed the action but with a different type of object (e.g., “go get the car” and the infant retrieved a different object) or performed an action that did not match with the target object (e.g., “build” and infants banged blocks), or coded as no correspondence when infants' action and mothers' verb input were unrelated. The findings showed that mothers say verbs that precisely map onto the action their infant is performing more often than they produce imprecise verbs or verbs that do not correspond to the current action.

Importantly, most of the verb labeling events in West et al. (2022) were driven by infants' actions—that is, mothers were sensitive to their infants' in the moment behaviors and produced verbs in response to an action (i.e., after the infant had started moving). Moreover, this and other work shows that, as infants hone their motor abilities, caregivers' verb input changes. For example, older infants receive more frequent *and* varied verb utterances from caregivers than younger infants do (West et al. 2022). When in motion, walking infants hear twice as many verbs describing locomotor actions (e.g., go, bring) than aged‐matched crawling infants (West et al. 2023).

Caregivers also increase the number of noun utterances describing objects as infants explore them (West and Iverson 2017). In turn, infants engage with objects for longer when their caregiver is responsive to their actions (McQuillan et al. 2020). In noun learning, experimental studies show that the co‐occurrence between infants' actions and caregiver labeling supports word learning. Infants' manipulations of objects help them to “declutter” the visual environment, making objects the focus of their attention which creates optimal moments for noun learning when paired with labeling (Pereira, Smith, and Yu 2014; Yoshida and Smith 2008; Yu and Smith 2012). Consequently, infants are more likely to learn the name for a novel object if it was labeled by their caregiver during these moments (Yu and Smith 2012). Later, nouns are more likely to be found in infants' vocabulary if they have experience manipulating the object (Suarez‐Rivera, Linn, and Tamis‐LeMonda 2022). It is plausible, therefore, that infants' actions on objects help to guide their noun learning. Infants can much more easily pare down the number of possible word meanings by manipulating an object, which guides attention to the referent during labeling.

Whether infants' motor actions are also associated with their verb acquisition in a similar way is currently untested. While the evidence reviewed suggests that gaining motor abilities increases the frequency of verb learning opportunities, no study has yet tested whether greater motor ability is associated with greater verb knowledge specifically. Here, we propose that the size of infants' receptive verb vocabulary is tightly linked with their motor abilities and that this link may in fact be stronger than the link with noun vocabulary. Why might the association between motor abilities and vocabulary size be different for verbs and nouns?

First, early nouns possess many advantages that early verbs do not. Nouns describe cohesive elements of the world that are easily individuated. In contrast, verbs describe actions and events that are ephemeral and relational by nature which makes them challenging to pinpoint in the world (Gentner 1982). This is evidenced by infants' ability to individuate objects as early as 4‐months‐old (e.g., Spelke et al. 1995) and their assumption that a new label refers to an unfamiliar object by 12‐months‐old (Pomiechowska et al. 2021). These findings suggest that infants can learn new nouns by extracting information readily available within the environment, and indeed 6‐ to 9‐months‐old already know several nouns (Bergelson and Swingley 2012; Tincoff and Jusczyk 1999, 2012). Further, an associative neural network, trained only on visual and audio recordings (i.e., no embodied input) captured from a child's point of view between the ages of 6‐ to 25‐months‐old, was able to learn several object‐word referents from visual and labeling experience alone (Vong et al. 2024). In contrast, infants begin individuating actions later, around 6‐months‐old (Sharon and Wynn 1998; Wynn 1996), and begin parsing them from continuous motion only by around 10‐months‐old (Baldwin et al. 2001). Compared to objects, it is challenging for infants to detect the boundaries of actions and determine which perceptual elements of the movement are relevant to a verb (Gleitman and Gleitman 1992). This contributes to children's frequent failures in mapping novel verbs onto new actions across early childhood (e.g., Imai et al. 2005, 2006, 2008). As such, children are thought to rely on several different, interacting mechanisms to overcome the challenge of identifying verb referents (Childers, Bottera, and Howard 2018). Self‐produced actions may support action identification by making it more salient compared to solely observing the actions of others (but see Childers et al. 2022). Possibly, motor behaviors cultivate opportunities that verb learning critically depends on, in ways that noun learning does not (to the same extent at least).

Second, many of children's early produced verbs describe actions involving bodily movements, which may suggest that verb learning is grounded in early sensorimotor experiences (Barsalou 2008; Glenberg and Gallese 2012; Piaget 1952). Children often first learn verbs that are tightly associated with specific parts of the body (e.g., *bite* with the mouth, *clap* with hands) before learning those that are not (e.g., *pretend*; Fenson et al. 1994; Huttenlocher, Smiley, and Charney 1983; Maouene, Hidaka, and Smith 2008, 2011). Bodily verbs are inherently more concrete, with opportunities to learn these verbs driven by self‐action and by observing the actions of others. In addition, self‐produced actions may be particularly important during early verb learning. For example, infants better understand a new action when they have first‐hand experience with that action compared to observational experience alone (Gerson and Woodward 2012, 2014a, 2014b). This action knowledge is later contained in infants' broader verb‐action concepts, contributing to their understanding of verb meanings (Sootsman Buresh, Woodward, and Brune 2006). That is, infants' active experience with motor actions helps them to form complex action representations and recognize contingencies between their own motor actions and action consequences, as well as common co‐occurrences with those actions (e.g., verb labels).

Video‐corpus data of toddlers and their caregivers interacting during daily activities show that young children primarily produce verbs that describe their current actions rather than the actions of others (Huttenlocher, Smiley, and Charney 1983). Laboratory‐based research with 2‐ and 3‐year‐old children has also shown that younger children benefit from motoric experience with a new action when it comes to understanding the meaning of a novel manner verb (Gampe, Brauer, and Daum 2016). In this study, children learned a new manner action paired with a novel verb. Children either saw the experimenter demonstrate the new action twice or saw the experimenter demonstrate the action once before performing the action themselves. Though 3‐year‐olds learned the new verb in both conditions, 2‐year‐olds only learned the new verb when they had active experience with the action too. Possibly, performing an action themselves could be more attention grabbing or make the action easier to encode than the actions of others (Huttenlocher, Smiley, and Charney 1983). For example, self‐producing an action may make it more “concrete” or tangible through rich multisensory information provided by the body (De Klerk, Filippetti, and Rigato 2021) and, thus, allow for a more transparent verb mapping.

## 4 Current Study

We sought to explore associations between infants' parent‐reported motor abilities and concurrent vocabulary size between the ages of 6 and 24 months. Between these ages, infants' motor and language skills undergo substantial change, enabling exploration of how associations between these domains may vary across development. At 6 months, infants have just begun understanding their first nouns (Bergelson and Swingley 2012; Tincoff and Jusczyk 1999, 2012) and, from this point steadily acquire new words (Carey 1978) until experiencing a sudden increase in vocabulary around 18–24 months (i.e. the vocabulary spurt; Goldfield and Reznick 1990). In the motor domain, by 4‐ to 6‐months‐old, infants can typically produce intentional reaching actions, and learn to sit without support. Over time, they learn to carefully co‐ordinate their hand movements, stand, walk and run around without assistance (Adolph and Robinson 2015), typically reaching these motor milestones between 12 and 18 months.^2^

Previous studies have focused on broad language outcomes by examining total vocabulary size. We aimed to examine whether infants' motor abilities are differentially associated with their verb and noun understanding. The link between motor actions and vocabulary development (in general) and the benefits of motor actions on noun learning are both well established. As such, we expect that both infants' verb *and* noun vocabulary sizes will be associated with their motor development. However, as we propose that self‐produced actions are especially important for verb over noun learning, we hypothesize that this relation will be significantly stronger for verbs. As productive vocabulary—especially for verbs—is relatively limited across this age range, we focused our exploration on receptive language only. In our primary analysis, we focus on infants' overall motor development cross‐sectionally to explore links between motor development and verb and noun comprehension, as we posit that greater motor ability (in general) enables infants to perform new types of actions that their parents can label with verbs. However, as many previous studies have reported links between gross and/or fine motor skills and vocabulary acquisition, we also report analyses for gross and fine motor skills separately.

## 5 Method

### 5.1 Ethical Approval

The present study was conducted according to guidelines laid down in the Declaration of Helsinki. Informed consent was obtained from the responding caregiver prior to completing the survey. This study is associated with ethics application number EC.19.03.12.5616A and was approved by the School of Psychology Ethics Committee at Cardiff University, UK.

## 6 Participants

Eighty‐three caregivers anonymously completed an online survey regarding their infant aged between 6 and 24 months (52 female infants, *M* = 16 months, *SD* = 4.97 months, *range* = 6.66–23.80 months). Each caregiver provided data for one infant.^3^ Of the 83 infants, 18 were aged 6‐ to 11‐months‐old, 32 were between 12‐ and 18‐months‐old, and 33 were aged 18‐ to 24‐months‐old. Sample size was determined on the basis of previous work (Gonzalez, Alvarez, and Nelson 2019). This sample size is sensitive enough to detect correlation coefficients of 0.30 and above at 80% power. Families were recruited via social media posts, email invites via a developmental database, or via a local health and care research newsletter, Healthwise Wales. Healthwise Wales is a Health and Care Research Wales initiative, which is led by Cardiff University in collaboration with SAIL, Swansea University (Hurt et al. 2019; Jones et al. 2014; Townson et al. 2020). All infants were monolingual, English‐hearing infants (i.e., heard English 75% or more at home or in care settings; Bergelson and Swingley 2012, 2013, 2015). All infants were born full term (i.e., 37 weeks or later) and were reported as typically developing with no known developmental delays. Seventy‐six families were based in Wales and seven were based in England. Details about infant and caregiver ethnicity were unfortunately not collected.

An additional 58 parents responded but were excluded due to their infant hearing less than 75% English at home (*n =* 19), having a history of developmental delays or developmental disorders (*n =* 6), premature birth (*n =* 5), experiencing language difficulties or delay (*n* = 1), were diagnosed with hearing loss (*n =* 4), or due to not completing the measures (i.e., having high levels of missing data; *n =* 23). Exclusion details due to missing data are described in more detail in the Data Preparation section.

The average age of the responding caregiver was 32 years (*SD* = 4.84 years, *range* = 22–44 years). Thirteen parents (14.4%) were educated up to high school equivalent level (i.e., GCSE or A‐Level qualification), 7 had a vocational qualification (8.4%), 41 (49.4%) had Bachelor's degrees, 14 (16.9%) had Master's degrees, and 8 (9.6%) had an MD, PhD, or equivalent.

## 7 Parent‐Report Measures

Caregivers completed all measures through an anonymous online survey hosted by REDCap (Research Electronic Data Capture; Harris et al. 2009, 2019).

### 7.1 Oxford Communicative Development Inventory—Extended Version

The extended O‐CDI (Hamilton, Plunkett, and Schafer 2000) is a parent report measure of vocabulary size, used with infants up until 26 months. It includes a total of 552 word items. Caregivers indicate on a checklist whether their infant understands or produces each word, yielding scores for both production and comprehension. We also gave an additional response option of “does not understand” to differentiate between missing data and non‐comprehension. We reminded parents that the O‐CDI is appropriate for a broad age range and that there may be words that their baby did not yet understand or say.

Our hypotheses were centered around verb and noun items. CDIs, including the O‐CDI, often do not include verbs that describe some of the earliest actions/motor skills/gestures infants learn perform (e.g., clap, crawl, wave). In contrast, they include many noun items describing the objects infants first manipulate (e.g., blocks, cup, banana). As we reasoned that infants' motoric experiences with relevant actions would be important for their verb learning, we added verbs describing some of these early learned actions. We added 17 verb items to the 69 existing verb items; *brush, build, clap, crawl, dig, fly, lick, nod, pat, point, pour, shake, sit, sniff, spit, talk,* and *wave*. These verbs were derived from items in standardized measures of gesture, action and motor abilities that children typically learn to perform during early development: the Early Motor Questionnaire (EMQ; Libertus and Landa 2013) and MacArthur–Bates: Actions and Gestures (M‐CDI; Fenson et al. 1994). This resulted in a total of 86 verbs.^4^ For nouns, 359 nouns were counted in the O‐CDI.^5^

For correlational analyses, we computed word comprehension scores by summing the total number of words understood. Production scores were not explored in this study. For verbs, scores could total 86. For nouns, scores could total 359. For all items in the O‐CDI, including verbs and nouns as well as animal sounds, adjectives, prepositions, question words, pronouns, and quantifiers, scores could total 568.^6^

### 7.2 Early Motor Questionnaire

Motor development was assessed using the EMQ (Libertus and Landa 2013), a parent‐report measure of early motor skills that can be used with infants up until the age of 24 months. The EMQ is divided into three sections organized by motor skill types with a total of 128 items: gross motor skills (49 items), fine motor (48 items), and perception action abilities (i.e., perceptual and sensory skills; 31 items). Caregivers respond using a five‐point scale that ranges from −2 to +2, indicating how certain they are that they have witnessed their child producing a given motor skill (e.g., perception action section; while sitting on your lap or fully supported in a high chair or car seat, have you noticed your child orient to noises and visually search for the cause of the noise?). A rating of −2 is given when the caregiver is certain that their child has not or cannot complete a motor skill and +2 is used when a caregiver remembers a specific instance when they witnessed their child using a motor skill. Caregivers can indicate they are uncertain whether their child can complete a motor skill by using a rating of 0, which they are encouraged to use sparingly. The EMQ has high convergent and concurrent validity, with EMQ scores correlating highly with standardized, examiner‐administered assessments of early motor development (e.g., Mullen Scales of Early Learning; Libertus and Landa 2013).

Scores are calculated by summing together responses for each given section. Total motor scores are summed across all questions with scores ranging from −256 to 256. The analyses presented below use infants' total scores, as well as gross and fine motor scores.

## 8 Data Preparation

Prior to scoring, we checked for missing values. Twenty‐three participants were identified to have disproportionate missing data, due to not completing the measures in full. These participants either did not complete any items or failed to complete most items on the Oxford‐CDI and/or the EMQ and, thus, were excluded from analyses. These participants on average had 272 missing items (*SD =* 212) for the Oxford‐CDI and had 56 missing items (*SD* = 59.5) for the EMQ.

Participants included in analyses had data missing for no more than four items on either the Oxford‐CDI or EMQ. On average, the final sample had missing data for 0.82 items on the Oxford‐CDI (*SD* = 1.11) and 0.43 items on the EMQ (SD = 0.83). Any individual missing data points in the final sample were replaced with zero. For CDI items, a score of zero indicates not understanding or saying an item and non‐completed items are typically treated as such (Hamilton, Plunkett, and Schafer 2000). For the EMQ, a score of zero indicates that parents are uncertain whether their child can perform a given action and, similarly, skipped items are typically replaced with zero in analyses (Libertus and Landa 2013).

### 8.1 Analysis Plan

The data and analysis scripts are publicly available on the OSF at https://doi.org/10.17605/OSF.IO/ZWY5K. All analyses were conducted in R (Version 4.4.1). We first aimed to conduct preliminary analyses to confirm expected positive associations between infants' age and their motor skills. We also wanted to investigate associations between age and receptive vocabulary size for verbs, nouns, and all words included in the O‐CDI. These analyses would ensure that links between age and motor/language development were observed, as would be expected in typically developing infants and demonstrating that age should be controlled for in subsequent analyses. Most studies exploring links between motor and language have primarily measured infants' total vocabulary size. We replicate this analysis approach here whilst also exploring whether this relation holds for verb and noun vocabulary separately (with and without age controlled for). We first conducted these correlations with total motor scores and then with gross and fine motor scores separately. Correlations were conducted with *cor_test*() function from the *rstatix* package (Kassambara 2023) and partial correlations were conducted with *pcor.test*() from the *ppcor* package (Kim 2015).

In our main analysis, we aimed to examine whether the association between motor skills and word comprehension is different for verbs and nouns. We fit a binomial generalized linear mixed‐effect model (GLMM) with a logit link function (i.e., a logistic mixed effect model) from the *lme4* package (glmer function; Bates et al. 2015). Logistic mixed effects models are especially advantageous for analyzing categorical language outcomes (e.g., CDI item responses), which cannot be interpreted using General Linear Models (GLMs; even when transformations such as arcsine‐square‐root are applied, Jaeger 2008). Logistic models aim to estimate the probability of a dichotomous outcome (understanding or not understanding a word) given the input variables (motor skills and word type). When using a logistic mixed effects model, categorical outcomes can be entered in their raw by‐item form and we used this analytical approach to analyze the item‐level dichotomous CDI comprehension scores. Such models enable the exploration of relations and interactions between fixed effects (predictors) and categorical outcome variables, whilst accounting for control variables (e.g., age). Mixed effect models extend traditional GLMs through the inclusion of random effects which accounts for individual differences within the model, such as participant and item effects.

Using this analytic approach, we aimed to test whether the relation between infants' motor skills and word comprehension differs for verbs and nouns, whilst controlling for age and accounting for participant and word item effects. Only responses to verb and noun items are entered into the model. Word comprehension is modeled as a binary dependent variable (0: *does not understand*, 1: *understands*).^7^ We include motor skills (total EMQ scores) and word type (verb | noun) as predictors. To ascertain whether the relation between infants' motor ability and word comprehension differed for verbs and nouns, we include an interaction between motor skills and word type. The model controls for infants' age, by including it as a fixed effect. We fit a maximal random effects structure (Barr et al. 2013). We include infants as random intercepts with by‐infant random slopes for word type, and word items as random intercepts with by‐word random slopes for motor skills and age. A random intercept only model (infants and word items) and a simple random effects structure model (infants and word items as random intercepts with by participant random slopes for word type) are also fit. Model comparisons revealed that the maximal random effects structure significantly improved the model fit (see Supplementary Materials). Continuous fixed effects are centered and scaled using the *scale*() function to address collinearity between EMQ scores and infants' age. Word type is sum contrast coded (−0.5: verb, 0.5: noun).^8^ Confidence intervals are computed with the *confint*() function. In the Results section, we report estimated likelihood of understanding a word which is often referred to as predicted probabilities in the literature. The estimated model had the following *lme4* structure:

WordComprehension∼EMQScore∗WordType+InfantAge+(1+WordType|Infants)+(1+EMQScore+InfantAge|WordItems)

We also re‐ran this analysis with infant sex and parental education as additional control variables. Finally, we conducted these analyses separately with fine and gross motor skills as predictors (rather than total EMQ scores). Each model controlled for the other type of motor skill scores (i.e., when gross motor skills were a predictor, fine motor skills were included as a control variable and vice versa) as well as controlling for age. These models used the following *lme4* structures:

**Gross Motor Skills:**

WordComprehension∼GrossEMQScore∗WordType+InfantAge+FineEMQScore+(1+WordType|Infants)+(1+GrossEMQScore+InfantAge|WordItems)

**Fine Motor Skills:**

WordComprehension∼FineEMQScore∗WordType+InfantAge+GrossEMQScore+(1+WordType|Infants)+(1+FineEMQScore+InfantAge|WordItems)

## 9 Results

### 9.1 Preliminary Analyses

**FIGURE 1:**
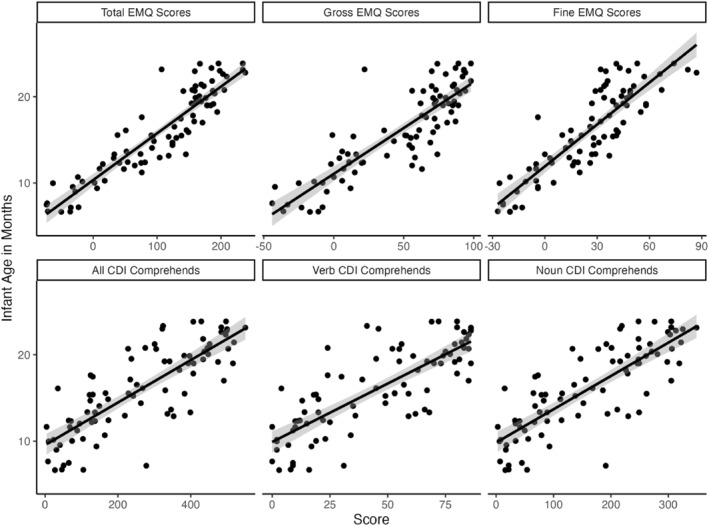
Associations between age, motor skills, and vocabulary scores. Ribbons represent standard error. *x*‐axis scale varies by measure: total EMQ scores, gross motor EMQ scores, fine motor EMQ scores, total O‐CDI score, verb O‐CDI score, or noun O‐CDI score. *y*‐axis shows infant age in months.

Descriptive statistics are reported in Table 1. O‐CDI scores produce count data outcomes that do not adhere to parametric assumptions (i.e., data is discrete and cannot produce values below zero) and visual inspections of all distributions revealed that age, EMQ scores, and receptive vocabulary scores deviated from normality, supported by Shapiro–Wilk tests (*p <* 0.001). As such, we used non‐parametric Spearman's rank correlations for all correlations and partial correlations. All tests were two‐tailed. Statistical significance was assessed at an *α* of 0.05 and Bonferroni corrections were applied to *p‐*values to correct for multiple comparisons.^9^ A strong positive correlation emerged between age and total EMQ scores, as well as between age and gross motor EMQ scores, and age and fine motor EMQ Scores (see Figure 1; *r*_s_ = 0.90, *p* < 0.001; *r*_s_ = 0.86, *p* < 0.001; *r*_s_ = 0.85, *p* < 0.001). Age was also positively associated with infants' total, verb, and noun comprehension scores, (*r*_s_ = 0.80, *p* < 0.001; *r*_s_ = 0.79, *p* < 0.001; *r*_s_ = 0.80, *p* < 0.001), respectively; showing that motor development and vocabulary development were strongly related to infants' age. As such, age was controlled for in all subsequent analyses.^10^

**TABLE 1:** Descriptive statistics for motor skill and vocabulary scores.

		Mean score	SD	Range
O‐CDI	All words comprehends	262.41	163.62	4–550
	All words says	80.66	115.68	0–466
	Verbs comprehends	45.06	29.07	0–86
	Verbs says	8.51	18.15	0–83
	Nouns comprehends	159.98	103.76	3–349
	Nouns says	53.35	76.50	0–295
EMQ	Total score	104.36	83.96	−73 to 238
	Fine motor score	25.13	26.53	−27 to 87
	Gross motor score	46.47	40.31	−44 to 98

*Note:* O‐CDI scores refer to the number of words reported to be understood and the number of words reported to be said; scores are reported for all items in the measure, verb items, and noun items. EMQ total scores and subscale scores (fine and gross motor skills) are reported. CDI production scores are reported for completeness, although only CDI comprehension scores are included in analyses.

## 10 Associations Between Motor Skills and Vocabulary Size

Correlations revealed strong positive associations between total motor skills and verb comprehension scores (*r*_s_ = 0.82, *p* < 0.001), noun comprehension scores (*r*_s_ = 0.83, *p* < 0.001), and total comprehension scores (*r*_s_ = 0.83, *p* < 0.001). These associations remained significant after controlling for age. Partial correlations revealed significant positive associations between motor skills and comprehension scores: verb comprehension scores (*r*_s_ = 0.39, *p* < 0.001), noun comprehension scores (*r*_s_ = 0.40, *p* < 0.001), and total comprehension scores (*r*_s_ = 0.40, *p* < 0.001).

Similar strong positive correlations were also revealed between fine motor skills and verb comprehension scores (*r*_s_ = 0.79, *p* < 0.001), noun comprehension scores (*r*_s_ = 0.81, *p* < 0.001), and total comprehension scores (*r*_s_ = 0.81, *p* < 0.001). These associations remained significant after controlling for age. Partial correlations revealed significant positive associations between fine motor skills and comprehension scores: verb comprehension scores (*r*_s_ = 0.36, *p* = 0.004), noun comprehension scores (*r*_s_ = 0.40, *p* < 0.001), and total comprehension scores (*r*_s_ = 0.39, *p* = 0.001).

**FIGURE 2:**
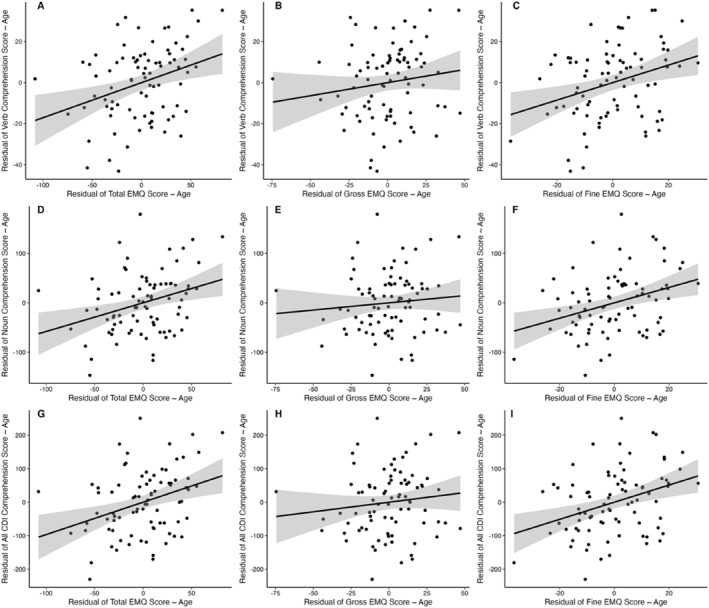
Associations between motor skills and vocabulary scores, controlling for age. Partial correlations between motor scores and number of words understood, controlling for age. Residuals from the relation between EMQ scores (total scores, gross motor scores, fine motor scores) and age are plotted on the *x* axis and residuals from the relation between CDI scores (verbs, nouns, all words) and age are plotted on the *y* axis. Ribbons represent standard error.

Strong positive correlations were also found between gross motor skills and verb comprehension scores (*r*_s_ = 0.73, *p* < 0.001, noun comprehension scores (*r*_s_ = 0.73, *p* < 0.001), and total comprehension scores (*r*_s_ = 0.73, *p* < 0.001). However, none of the associations between gross motor skills and word comprehension scores remained significant after controlling for age in partial correlations: verb comprehension scores (*r*_s_ = 0.17, *p* > 0.05), noun comprehension scores (*r*_s_ = 0.15, *p* > 0.05), and total comprehension scores (*r*_s_ = 0.14, *p* > 0.05). See Figure 2 for all correlation plots.

## 11 Are Motor Skills Associated More Strongly With Verb than With Noun Comprehension?

**FIGURE 3:**
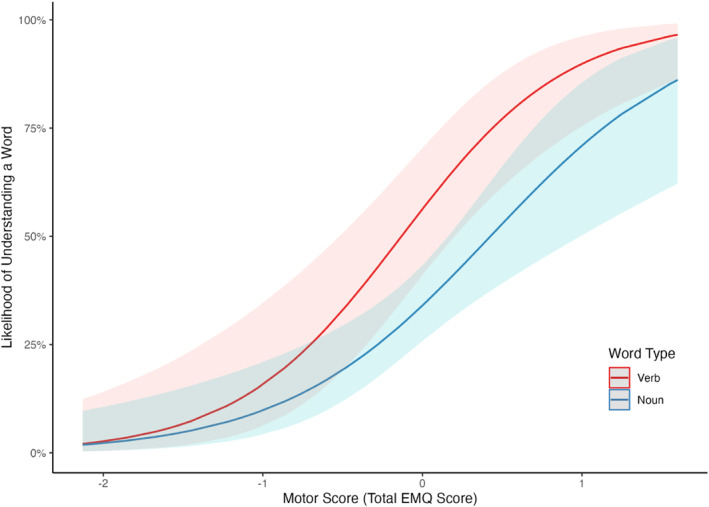
Results from logit mixed effects model showing an interaction between word comprehension, motor skills, and word type. On the *y* axis, is the likelihood of understanding a given word, ranging from 0% to 100%. On the *x* axis is the total EMQ score (centered and scaled). Ribbons represent 95% confidence intervals.

The fixed effects from the logistic mixed effect model, exploring the association between motor skills and word comprehension for verbs and nouns, are described in Table 2 and the random effects are described in Table 3. Crucially, the model revealed a significant interaction between motor skills and word type (*β* = −0.37, CI 95% [−0.65, −0.09], *SE* = 0.14, *z* = −2.62, *p* = 0.009), suggesting that the relation between motor skills and word comprehension differed for verbs and nouns. We followed up this interaction using the *emtrends*() function from the *emmeans* package (Lenth, 2024), which compares whether slopes, for each level of a categorical predictor, are statistically different from each other in models with significant interactions between a categorical and continuous predictor. The results showed that the association between word comprehension and motor skills was significantly greater for verbs than for nouns (*estimate* = 0.465, CI 95% [0.117, 0.812], *SE* = 0.177, *z* = 2.62, *p* = 0.009, see Figure 3). That is, for an infant of average age in our sample, motor skills were more strongly linked to the proportion of verbs comprehended than to the proportion of nouns comprehended.

**TABLE 2:** GLMM model results: Fixed effects.

	Model summary			
	*Β*	*SE*	*z*	*p*
Intercept	−0.20	0.22	−0.92	0.356
Total EMQ scores	1.74	0.41	4.22	< 0.001***
Word type	−0.92	0.28	−3.22	0.001**
Infant age	0.72	0.40	1.78	0.075
Total EMQ scores:word type	−0.37	0.14	−2.62	0.009**

*Note:* *p < 0.05; **p < 0.01; ***p < 0.001.

**TABLE 3:** GLMM model results: Random effects.

		Variance	Standard deviation
Word items	Intercept	4.67	2.16
	Total EMQ scores	0.08	0.27
	Infant age	0.11	0.33
Infants	Intercept	2.59	1.61
	Word type	0.92	0.96

The results also revealed a significant main effect of motor skills on word comprehension, with the likelihood of understanding a word increasing with greater motor ability (*β* = 1.74, CI 95% [0.93, 2.55], *SE* = 0.41, *z* = 4.21, *p* < 0.001). A significant main effect of word type also emerged (*β* = −0.91, CI 95% [−1.47, −0.36], *SE* = 0.28, *z* = −3.21, *p* = 0.001), reporting that the infant of average age and EMQ score in the sample understood a greater proportion of the verbs (52.3%) than they did nouns (43.7%). The main effect of age was not significant (*β* = 0.72, CI 95% [−0.07, 1.51], *SE* = 0.40, *z* = 1.78, *p* = 0.075^11^). We also ran an exploratory analysis with parent education and infant sex as additional control variables. The pattern of results remained the same across fixed effects. Parent education and infant sex were not significant predictors (see Supplementary Materials and OSF for details).

### 11.1 Robustness Checks

We conducted two additional analyses to assess the robustness of the interaction effect between total EMQ scores and word type. First, we also refitted the model in Tables 2 and 3 to a subset of the data that only included the original O‐CDI verb and noun items. We wanted to ascertain whether including additional verbs, that may be comprehended earlier in infancy, biased the results. The analysis revealed the same pattern of results across fixed effects and interactions (see Supplementary Information and OSF for details), indicating the addition of new verb items was not driving the interaction effect.

We also checked whether the imbalance in the number of noun versus verb items in our vocabulary checklist could account for the interaction we observed. As previously highlighted, CDIs contain a greater number of nouns than verbs. We partially addressed this difference in the number of word items for each word type by adding additional verbs into the CDI, representing actions and gestures children learn to perform across early development. Nonetheless, the number of verb and noun items remains heavily unbalanced (86 verbs, 359 nouns). To address this, we used a simulation approach to fit models with equal numbers of verb and noun items. We ran 500 logistic mixed effects models using the same *lme4* structure with each model using a randomly selected subset of nouns equal to the number of verbs (86 items each). For each model, we then extracted the motors skills by word type interaction estimate. Forty‐seven models (9.4%) failed to converge (i.e., produced un‐estimatable or unreliable model estimates) and/or had a singular fit (i.e., some parameters within the variance‐covariance estimated at zero) and were not included in the following analyses. Of the remaining 453 models, 80.3% (364) of the models revealed a significant interaction between motor skills and word type, with all estimates pointing in same direction as our primary analysis. We computed the mean interaction estimate across models and tested whether the mean interaction estimate was significantly different from zero using a one‐sample *t*‐test. Visual inspections of the distribution and a Shapiro‐Wilk test confirmed that the data were normally distributed. The results showed that the mean interaction estimate (*M* = −0.372) was significantly different from zero (*t*(452) = −98.598, *p* < 0.001). These results suggest that the interaction between motor skills and word type robustly held across models that included an equal number of verb and noun items and that the interaction was not due to the unbalance in number of word items.

## 12 Are Gross and Fine Motor Skills Associated More Strongly With Verb than With Noun Comprehension?

Finally, we explored whether gross and fine motor skills are separately associated with verb and noun comprehension. The fixed effects and random effects are described for the gross motor model in Tables 4 and 5 (respectively) and for the fine motor model in Tables 6 and 7 (respectively).

**TABLE 4:** Gross GLMM model results: Fixed effects.

	Model summary			
	*β*	*SE*	*z*	*P*
Intercept	−0.19	0.22	−0.87	0.386
Gross EMQ scores	0.21	0.36	0.57	0.567
Word type	−0.91	0.28	−3.20	0.001**
Infant age	1.06	0.37	2.85	0.004**
Fine EMQ scores	1.19	0.35	3.37	0.001**
Gross EMQ Scores:Word type	−0.34	0.13	−2.54	0.011*

*Note:* *p < 0.05; **p < 0.01; ***p < 0.001.

**TABLE 5:** Gross GLMM model results: Random effects.

		Variance	Standard deviation
Word items	Intercept	4.70	2.16
	Gross EMQ scores	0.00	0.05
	Infant age	0.18	0.42
Infants	Intercept	2.55	1.60
	Word type	0.91	0.95

**TABLE 6:** GLMM model results: Fixed effects.

	Model summary			
	*β*	*SE*	*z*	*P*
Intercept	−0.21	0.22	−0.93	0.354
Fine EMQ scores	1.44	0.36	3.96	< 0.001***
Word type	−0.91	0.29	−3.20	0.001**
Infant age	1.05	0.37	2.79	0.005**
Gross EMQ scores	0.02	0.35	0.07	0.948
Fine EMQ Scores:Word type	−0.37	0.14	−2.60	0.009**

*Note:* *p < 0.05; **p < 0.01; ***p < 0.001.

**TABLE 7:** GLMM model results: Random effects.

		Variance	Standard deviation
Word items	Intercept	4.70	2.17
	Fine EMQ scores	0.10	0.32
	Infant age	0.11	0.33
Infants	Intercept	2.59	1.61
	Word type	0.92	0.96

### 12.1 Gross Motor Skills

**FIGURE 4:**
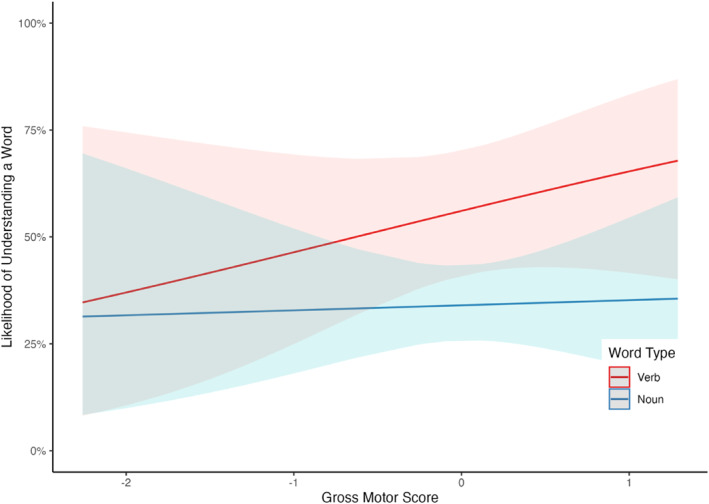
Results from logit mixed effects model showing an interaction between word comprehension, gross motor skills, and word type. On the *y* axis, is the likelihood of understanding a given word, ranging from 0% to 100%. On the *x* axis is the gross motor EMQ score (centered and scaled). Ribbons represent 95% confidence intervals.

This model revealed a significant interaction between motor skills and word type (*β* = −0.34, CI 95% [−0.60, −0.08], *SE* = 0.13, *z* = −2.54, *p* = 0.011), with verb comprehension being more strongly associated with gross motor skills compared to noun comprehension (*estimate* = 0.34, CI 95% [0.078, 0.604], *SE* = 0.134, *z* = 2.54, *p* = 0.011, see Figure 4. The main effect of gross motor skills was not significant (*β* = 0.21, CI 95% [−0.50, 0.91], *SE* = 0.36, *z* = 0.57, *p* = 0.567), but both control variables were: The model revealed significant main effects for fine motor skills (*β* = 1.19, CI 95% [0.50, 1.88], *SE* = 0.35, *z* = 3.37, *p* = 0.001) and age (*β* = 1.06, CI 95% [0.33, 1.79], *SE* = 0.37, *z* = 2.85, *p* = 0.004), with likelihood of understanding a word increasing as fine motor skills and age increased. The main effect of word type was also significant, with infants on average understanding a greater proportion of verbs than nouns (*β* = −0.91, CI 95% [−1.47, −0.35], *SE* = 0.28, *z* = −3.20, *p* = 0.001).

### 12.2 Fine Motor Skills

**FIGURE 5:**
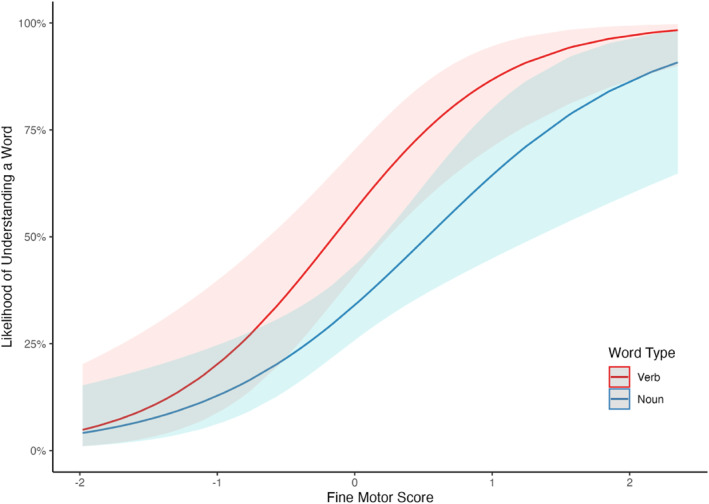
Results from logit mixed effects model showing an interaction between word comprehension, fine motor skills, and word type. On the *y* axis, is the likelihood of understanding a given word, ranging from 0% to 100%. On the *x* axis is the fine motor EMQ score (centered and scaled). Ribbons represent 95% confidence intervals.

This model also revealed a significant interaction between motor skills and word type (*β* = −0.37, CI 95% [−0.65, −0.09], *SE* = 0.14, *z* = −2.60, *p* = 0.009), with verb comprehension being more strongly associated with fine motor skills compared to noun comprehension (*estimate* = 0.373, CI 95% [0.092, 0.654], *SE* = 0.143, *z* = 2.60, *p* = 0.009, see Figure 5. The model also revealed significant main effects for fine motor skills (*β* = 1.44, CI 95% [0.73, 2.16], *SE* = 0.36, *z* = 3.96, *p* < 0.001) and age (*β* = 1.04, CI 95% [0.31, 1.78], *SE* = 0.37, *z* = 2.79, *p* = 0.005), with likelihood of understanding a word increasing as fine motor skills and age increased. As with previous models, the main effect of word type was significant, with infants on average understanding a greater proportion of verbs than nouns (*β* = −0.91, CI 95% [−1.47, −0.35], *SE* = 0.28, *z* = −3.20, *p* = 0.001). The main effect of gross motor skills was not significant (*β* = 0.02, CI 95% [−0.66, 0.71], *SE* = 0.35, *z* = 0.07, *p* = 0.948).

## 13 Discussion

We aimed to investigate whether infants' motor abilities are differentially associated with their concurrent verb and noun understanding, in a sample of infants aged 6‐ to 24‐months‐old. Replicating much previous work (e.g., Alcock and Krawczyk 2010; Choi et al. 2018; Clearfield 2011; Gonzalez, Alvarez, and Nelson 2019; He, Walle, and Campos 2015; Houwen et al. 2016; Karasik, Tamis‐Lemonda, and Adolph 2014; Oudgenoeg‐Paz, Volman, and Leseman 2012; Pereira, Smith, and Yu 2014; Schneider and Iverson 2022; Schroer and Yu 2022; Suarez‐Rivera, Linn, and Tamis‐LeMonda 2022; Valla et al. 2020; Walle and Campos 2014; West and Iverson 2021; Yu and Smith 2012), we first showed that the number of verbs and nouns infants were reported to understand increased as infants' overall motor skills developed, above and beyond the effect of age. We found the same pattern for fine motor skills, while gross motor skills were not associated with vocabulary once age was controlled for. Crucially, we also found an interaction between motor development and word comprehension, revealing that motor skills were more strongly linked with verb comprehension compared to noun comprehension—this interaction was significant for overall motor skills as well as fine and gross motor skills. Follow up simulation analyses demonstrated that this effect is robust, holding across models where an equal number of verbs and nouns were included by randomly subsampling nouns, suggesting that the interaction between word type and motor skills is unlikely to be driven by unbalanced numbers of verb and noun items in our CDI instrument. This suggests that the cascading impact of motor skills on word learning is likely not uniform across lexical acquisition. This is not to suggest that gains in motor skills are not important for noun comprehension; in fact our data also show that noun understanding is tightly linked to motor acquisition. Yet, the acquisition of motor skills may play a more important role in verb, compared to noun, learning. Below we discuss possible explanations for this finding.

Descriptions of early vocabularies show that they are typically noun‐dominated in English (Gentner 1978). Similarly, several lab studies show that children struggle to learn novel verbs (e.g., Childers and Tomasello 2002; Imai, Haryu, and Okada 2005 2008; Kersten and Smith 2002). Theoretical approaches to word learning suggest that this disparity is not the consequence of intrinsic differences between word classes *per se*, but rather because verbs describe abstract concepts that have variable meanings across languages (Gentner 1978, 2006; Gentner and Boroditsky 2001). Given these challenges, it is likely that children exploit several word learning mechanisms and environmental cues to ascertain the meaning of a given verb (Gillette et al. 1999; Gleitman 1990; Naigles 1990; Samuelson and Smith 1998; Tomasello 1992b, 1995). Advancing motor skills may serve as one such mechanism. Supporting evidence comes from West et al. (2022), who demonstrated that caregivers frequently respond to infants' actions with congruent verbs. Caregivers of older infants (18‐month‐olds), with larger motor and linguistic repertoires, were found to respond to their infants' actions more frequently and with a greater diversity of verbs than caregivers of younger infants (13‐month‐olds). Thus, infants' actions and their increasing ability to perform actions may bolster their verb learning by shaping the frequency and variability of verbs they hear from caregivers.

Further, active experience performing relevant actions across verb naming exposures may also help organize infants' action concepts. Active experience with actions helps infants to recognize actions as intentional and goal‐directed (Gerson and Woodward 2014a, 2014b). Performing verb‐relevant actions may support infants' understanding of which components of a given action are conveyed in a verb (Sootsman Buresh, Woodward, and Brune 2006). An alternative (but not mutually exclusive) possibility is that performative action experience makes the action more salient. Note we are not suggesting that motoric experience with relevant actions is necessary for verb learning (see Iverson 2010), but rather that it may serve as an additional, supportive mechanism through which infants can reduce the number of potential referents for a verb.

Though research shows infants' actions are related to their noun learning, our findings suggest that motor skills may play a smaller role during noun than verb acquisition. For example, research shows infants' object holding and head positioning supports their novel noun learning when it co‐occurs with parental labeling (Pereira, Smith, and Yu 2014; Schroer and Yu 2022; Yu and Smith 2012). Infants' actions in these moments are thought to reduce referential uncertainty by providing infants with less cluttered visual scenes. Yet, nouns often describe concrete referents that infants find easy to individuate in the real world, and infants readily map a novel noun onto a recently familiarized object category from visual experience alone (Pomiechowska and Gliga 2019). The same cannot be said for verbs, with children younger than 5 years often failing to map a novel verb onto a recently seen action (e.g., Imai et al. 2005, 2006, 2008). For this reason, infants may be less dependent on their own actions to infer the meaning of novel nouns and may rely more heavily on them to grasp the meaning of novel verbs. It is also possible that infants' actions may be especially beneficial for learning more abstract or relational nouns. Future research could compare abstract nouns and verbs directly, and test whether concreteness and imaginability partially (or fully) drive the differences found in the current analyses (Ma et al. 2009; McDonough et al. 2011).

Whether the detected interaction between word comprehension and motor skill is driven by specific motor milestones that have been explored in the literature as key inflection points (e.g., walking, independent sitting, grasping) is unclear. We ran additional analyses that show that both increased gross and fine motor ability are more strongly associated with verb than noun comprehension but these findings cannot speak to whether specific gross and/or fine motor milestones are driving effects—though it is interesting to note that gross motor skills were not found to be predictive of word comprehension above and beyond the effect of age which seem to suggest fine motor skills play a more important role. We used global, composite scores of motor development (as well as global scores of gross and fine motor development) to capture motor development holistically but future research could explore whether these interactions may be associated with particular motor milestones.

Our analyses also revealed main effects in addition to the critical interaction between motor skills and word type. As expected, and confirming the correlational analyses, greater overall motor ability and fine motor ability were associated with increased likelihood of word comprehension (regardless of word type). Across all analyses, these relations held when age was controlled for. Embodied perspectives have long suggested that the cascading effects of motor development are not simply the result of maturation alone (Iverson 2010). Rather, developing motor skills bolster infants' practice of communicative abilities and re‐organizes their interactions with the world around them (Campos et al. 2000; Iverson 2010, 2022) creating rich word learning opportunities. In contrast, gross motor skills were not found to be associated with word comprehension when age (in both correlation and mixed model analyses) and fine motor skill (mixed model analyses only) was controlled for. This finding is somewhat unexpected, given that many studies have reported links between gross motor skills and comprehensive vocabulary during infancy (e.g., He, Walle, and Campos 2015; Valla et al. 2020; Walle and Campos 2014). However, these findings do align with other studies that have also reported a lack of association between gross motor ability and vocabulary (Karasik, Tamis‐Lemonda, and Adolph 2014) or that the strength of the association reduces or the association no longer holds once other covariates are controlled for such as other motor skills, gesture skills, and cognitive ability (Alcock and Krawczyk 2010; He, Walle, and Campos 2015; Houwen et al. 2016). For example, cross‐cultural work by He, Walle, and Campos (2015) found that walking ability was only associated with expressive vocabulary (but not receptive) in American infants, after controlling for infants' self‐produced locomotor experience. Whereas, for Chinese infants, the associations held for both expressive and receptive vocabulary. These findings possibly suggest that, given that only fine motor skills were a significant predictor (in both models), that fine motor skills may contribute to infants' word learning more so than gross motor skills. However, examinations of the fine and gross motor distributions in our sample show that fine motor skills were more evenly spread across the reported range (i.e., more variable) compared to gross motor skills, which may explain why gross motor skills were not found to significantly predict word comprehension.

In addition, we found an effect of word type: Infants in the sample understood, on average, a greater proportion of verbs than nouns. This finding is possibly explained by the difference in the number of verb compared to noun items included in the O‐CDI. Like most CDIs, the number of verb items included in the measure is much smaller than the number of nouns. This difference reflects the fact that children tend to learn many more nouns in their early development than verbs. It is possible that the average infant in the sample understood a greater proportion of verbs than nouns as the volume and variability of noun items was much greater than that of the verb items. It is worth noting that this may, in part, explain the interaction found between motor skills, word type, and word comprehension. That is, given that we had fewer verb items and many of the verb items (including those added) may be comprehended earlier in development, the results may have skewed the likelihood of understanding words “in favor” of verbs. Though this seems an unlikely explanation, given that the simulation analysis demonstrated that the interaction between motor skills and word type was significant across 80.3% of models where the number of verbs to nouns was equalized.

Finally, note that the effect of age was not significant in our primary logit mixed model, despite being linked with infants' vocabulary scores in preliminary correlations. This is likely due to multicollinearity between infants' EMQ scores and age—that is, infants' EMQ scores share overlapping variance with age (accordingly, age remains a significant predictor when EMQ scores are removed from the model; see OSF).

### 13.1 Limitations and Future Directions

The current study cannot speak to a causal relation between motor development and infants' language development. Experimental studies could investigate whether motoric training with novel actions paired with novel verbs, contrasted with passive observation (e.g., Gerson, Bekkering, and Hunnius 2015; Gerson and Woodward 2014b), better supports infants' verb learning (see Dargue, Sweller, and Jones 2019; de Nooijer et al., 2013; Gampe, Brauer, and Daum 2016, for similar studies with children and adults). Future work should also test whether verb input from caregivers during infants' congruent actions result in increased verb learning (Pereira, Smith, and Yu 2014; Schroer and Yu 2022; Suarez‐Rivera, Linn, and Tamis‐LeMonda 2022; Yu and Smith 2012).

We utilized parent‐report measures of motor and language development, which are validated, well‐studied, cost‐effective, and efficient. EMQ scores are correlated with examiner‐administered motor assessments (Libertus and Landa 2013) and CDI responses often align with infants' word comprehension measured in looking time studies (Styles and Plunkett 2009). Yet, parent‐report measures can be prone to over or under‐estimations of children's current capabilities (Houston‐Price, Mather, and Sakkalou 2007; Tomasello and Mervis 1994). Whilst these measures likely provide reasonable estimations of infants' development, future research should seek to corroborate these relations under carefully controlled conditions.

One possible limitation of our study is the use of a parent report measure of word comprehension, the O‐CDI. The O‐CDI, like other CDIs, asks parents to recollect instances where their infant demonstrated evidence of understanding a word. Parents may be most accurate at assessing their infants' knowledge of a verb when their infant is also able to perform that action (most likely in response to a parental request; for example, “Can you *give* that to Mummy?” before receiving an object from their infant). In contrast, when it comes to noun knowledge, parents may rely on different cues such as pointing or turning to gaze at an object after hearing it labeled. As such, parents of motorically advanced infants may have more cues (i.e., motor behaviors) available to aid in their assessment of their infants' verb knowledge than parents of less motorically advanced infants. If this were the case, then the interaction between motor abilities and word type could be—at least in part—explained by parents' enhanced ability to assess their infants' understanding of verbs specifically, rather than by the fact that more motorically advanced infants actually know proportionally more verbs than nouns. CDIs have been proven to be reliable in estimating children's word knowledge (and comparable to looking time measures; Styles and Plunkett 2009), but because they mostly contain nouns, we do not know if they are less reliable for verbs than nouns. To address this, future research could use more objective measures of word knowledge (e.g., looking‐while‐listening paradigms) to explore these relations.

This is the first study to explore how links between motor development and word comprehension may vary between nouns and verbs. However, we did not collect broader information about the sample's SES, ethnicity, and demographic information that would have provided insights into the representativeness of our sample. Previous research has shown that both language and motor development (and links between the two) can be influenced by other covariates such as SES (e.g., Dailey and Bergelson 2022; Tacke, Bailey, and Clearfield 2015), general cognitive ability (e.g., Alcock and Krawczyk 2010), as well as ethnicity and culture (e.g., Kuchirko and Tamis‐LeMonda 2019; Tamis‐Lemonda et al. 2012). Here, we found that parental education and infant sex did not predict infants' word comprehension but future research exploring links between motor development and different aspects of word comprehension should seek to also capture measures of SES, ethnicity, parental factors (e.g., age, education, employment), and infant cognition to explore the impact of contextual factors on these relations.

## 14 Conclusions

Studies of language development reveal strong associations between early motor ability and the number of words infants understand and produce. Such work has sparked discussions around the cascading influence of motor development on early cognition and language acquisition. Much of this research has focused on broad measures of children's vocabulary development by capturing the size of their receptive and productive lexicons. Here, we show that infants' verb understanding, in particular, is strongly tied to their motor ability, compared to noun understanding, during the first 2 years of life. Given that verb referents are abstract and challenging to identify in the world, infants may exploit experiences with self‐produced actions to ascertain the meaning of novel verbs.

## Author Contributions

**Kelsey L. Frewin:** conceptualization, formal analysis, funding acquisition, investigation, project administration, writing – original draft. **Sarah A. Gerson:** conceptualization, supervision, writing – review and editing. **Ross E. Vanderwert:** writing – review and editing. **Chiara Gambi:** conceptualization, formal analysis, supervision, writing – review and editing.

## Conflicts of Interest

The authors declare no conflicts of interest.

## Supporting information

Supplementary Material

## Data Availability

Our data and analysis scripts are publicly available on the Open Science Framework at https://doi.org/10.17605/OSF.IO/ZWY5K.

## Endnotes

^1^
Note that it is unclear whether infants and children formulate these concepts into such adult‐like definitions and categories of nouns and verbs. Indeed, sometimes object and action concepts are intertwined during the first 2 years of life (Hagihara et al. 2022) and children's verb and noun knowledge have been reported as interrelated during early development (e.g., noun knowledge during at 16‐months‐old predicting verb knowledge at 20‐months‐old, Longobardi et al. 2017; verb presence in a sentence supporting novel noun identification, Ferguson, Graf, and Waxman 2014; Goodman, McDonough, and Brown 1998). Throughout this manuscript we use the definitions of nouns and verbs that are common in adult language in the absence of a better alternative, but future work using experimental methods will need to be carried out to understand how these categories apply to infant vocabularies.

^2^
Motor milestones, approaches to motor development, and environments in which infants' develop their motor skills can vary cross‐culturally (Adolph and Hoch 2018; Adolph and Robinson 2015). The motor milestones described in this paragraph have been reported from western samples, and are thus considered relevant for our sample of infants living within the United Kingdom.

^3^
All caregivers were given the option of providing data for multiple children within this age range.

^4^
The original extended O‐CDI contained 70 verb items. Due to experimenter error, responses for the item *write* were not collected. Therefore, results in the described data included 69 of the original verb items, 17 added verbs and, thus, a total of 568 words in the checklist.

^5^
Several nouns describing internet phenomena (e.g., Facebook, Skype) were deemed as too abstract for the current sample and, thus, were not included in noun comprehension scores. This excluded seven items from noun comprehension scores.

^6^
Typically extended O‐CDI scores can total 569. However, responses for one item (i.e., *write*) were not collected due to experimenter error and, thus, scores were out of a total 568.

^7^
Items reported to be “said” are also included as understood items.

^8^
Sum contrast coding of categorical fixed effect interactions essentially “centers” the effect at the grand mean between the two groups and are easier to interpret than treatment coding (i.e., 0 | 1).

^9^
Bonferroni corrections describe an adjusted *p‐*value, rather than the use of an adjusted *α* level.

^10^
Controlling for age ensures the unique relation between motor and language development can be explored beyond the effect of age.

^11^
We interpret age to be a non‐significant predictor in this model due to the high correlation between age and EMQ (as reported in the Preliminary Analysis section). When we re‐conducted this model without EMQ as a predictor, age was a significant predictor (this analysis can be viewed on our OSF page).

## References

[infa12638-bib-0001] Adolph, K. E. , and J. E. Hoch. 2019. “Motor Development: Embodied, Embedded, Enculturated, and Enabling.” Annual Review of Psychology 70: 141–164. 10.1146/annurev-psych-010418. PMC6320716 30256718

[infa12638-bib-0002] Adolph, K. E. , and S. R. Robinson . 2015. “Motor Development.” In Handbook of Child Psychology and Developmental Science: *Cognitive Processes*, edited by L. S. Liben, U. Müller, and R. M. Lerner, 7th ed., 113–157. John Wiley & Sons, Inc. 10.1002/9780470147658.chpsy0204.

[infa12638-bib-0003] Alcock, K. J. , and K. Krawczyk . 2010. “Individual Differences in Language Development: Relationship With Motor Skill at 21 Months.” Developmental Science 13, no. 5: 677–691. 10.1111/j.1467-7687.2009.00924.x. 20712734

[infa12638-bib-0004] Antognini, K. , and M. M. Daum . 2019. “Toddlers Show Sensorimotor Activity During Auditory Verb Processing.” Neuropsychologia 126: 82–91. 10.1016/j.neuropsychologia.2017.07.022. 28734698

[infa12638-bib-0005] Baldwin, D. A. , J. A. Baird , M. M. Saylor , and M. A. Clark . 2001. “Infants Parse Dynamic Action.” Child Development 72, no. 3: 708–717. 10.1111/1467-8624.00310. 11405577

[infa12638-bib-0006] Barr, D. J. , R. Levy , C. Scheepers , and H. J. Tily . 2013. “Random Effects Structure for Confirmatory Hypothesis Testing: Keep it Maximal.” Journal of Memory and Language 68, no. 3: 255–278. 10.1016/j.jml.2012.11.001. PMC3881361 24403724

[infa12638-bib-0007] Barsalou, L. W. 2008. “Grounded Cognition.” Annual Review of Psychology 59, no. 1: 617–645. 10.1146/annurev.psych.59.103006.093639. 17705682

[infa12638-bib-0008] Bates, D. , M. Mächler , B. M. Bolker , and S. C. Walker . 2015. “Fitting Linear Mixed‐Effects Models Using lme4.” Journal of Statistical Software 67, no. 1: 48. 10.18637/jss.v067.i01.

[infa12638-bib-0009] Bergelson, E. 2020. “The Comprehension Boost in Early Word Learning: Older Infants Are Better Learners.” Child Development Perspectives 14, no. 3: 142–149. 10.1111/cdep.12373. 33569084 PMC7872330

[infa12638-bib-0010] Bergelson, E. , and D. Swingley . 2012. “At 6–9 Months, Human Infants Know the Meanings of Many Common Nouns.” Proceedings of the National Academy of Sciences 109, no. 9: 3253–3258. 10.1073/pnas.1113380109. PMC3295309 22331874

[infa12638-bib-0011] Bergelson, E. , and D. Swingley . 2013. “The Acquisition of Abstract Words by Young Infants.” Cognition 127, no. 3: 391–397. 10.1016/j.cognition.2013.02.011. 23542412 PMC3633664

[infa12638-bib-0012] Bergelson, E. , and D. Swingley . 2015. “Early Word Comprehension in Infants: Replication and Extension.” Language Learning and Development 11, no. 4: 369–380. 10.1080/15475441.2014.979387. 26664329 PMC4671511

[infa12638-bib-0013] Bornstein, M. H. , L. R. Cote , S. Maital , et al. 2004. “Cross‐Linguistic Analysis of Vocabulary in Young Children: Spanish, Dutch, French, Hebrew, Italian, Korean, and American English.” Child Development 75, no. 4: 1115–1139. 10.1111/j.1467-8624.2004.00729.x. 15260868

[infa12638-bib-0014] Campos, J. J. , D. I. Anderson , M. A. Barbu‐Roth , E. M. Hubbard , M. J. Hertenstein , and D. Witherington . 2000. “Travel Broadens the Mind.” Infancy 1, no. 2: 149–219. 10.1207/S15327078IN0102_1. 32680291

[infa12638-bib-0015] Carey, S. 1978. “The Child as Word Learner.” In Linguistic Theory and Psychological Reality, edited by M. Halle , J. Bresnan , and G. A. Miller , 264–293. Cambridge, MA: MIT Press.

[infa12638-bib-0016] Caselli, M. C. , E. Bates , P. Casadio , et al. 1995. “A Cross‐Linguistic Study of Early Lexical Development.” Cognitive Development 10, no. 2: 159–199. 10.1016/0885-2014(95)90008-X.

[infa12638-bib-0017] Childers, J. B. , A. Bottera , and T. Howard . 2018. “Verbs: Learning How Speakers Use Words to Refer to Actions.” In Early Word Learning, edited by G. Westermann and N. Mani , 70–82. London: Routledge. 10.4324/9781315730974.

[infa12638-bib-0018] Childers, J. B. , and M. Tomasello . 2002. “Two‐year‐olds Learn Novel Nouns, Verbs, and Conventional Actions From Massed or Distributed Exposures.” Developmental Psychology 38, no. 6: 967–978. 10.1037//0012-1649.38.6.967. 12428708

[infa12638-bib-0019] Childers, J. B. , E. Warkentin , B. M. Porter , M. Young , S. Lalani , and A. Gopalkrishnan . 2022. “Preschool Children’s Processing of Events During Verb Learning: Is the Focus on People (Faces) or Their Actions (Hands)?” Brain Sciences 12, no. 3: 344. 10.3390/brainsci12030344. 35326300 PMC8946060

[infa12638-bib-0020] Choi, B. , K. A. Leech , H. Tager‐Flusberg , and C. A. Nelson . 2018. “Development of Fine Motor Skills Is Associated With Expressive Language Outcomes in Infants at High and Low Risk for Autism Spectrum Disorder.” Journal of Neurodevelopmental Disorders 10, no. 1: 1–11. 10.1186/s11689-018-9231-3. 29649977 PMC5898056

[infa12638-bib-0021] Choi, S. , and A. Gopnik . 1995. “Early Acquisition of Verbs in Korean: A Cross‐Linguistic Study.” Journal of Child Language 22, no. 3: 497–529. 10.1017/S0305000900009934. 8789512

[infa12638-bib-0022] Clearfield, M. W. 2011. “Learning to Walk Changes Infants’ Social Interactions.” Infant Behavior and Development 34, no. 1: 15–25. 10.1016/j.infbeh.2010.04.008. 20478619

[infa12638-bib-0023] Clearfield, M. W. , C. N. Osborne , and M. Mullen . 2008. “Learning by Looking: Infants’ Social Looking Behavior Across the Transition From Crawling to Walking.” Journal of Experimental Child Psychology 100, no. 4: 297–307. 10.1016/j.jecp.2008.03.005. 18452944

[infa12638-bib-0024] Dailey, S. , and E. Bergelson . 2022. “Language Input to Infants of Different Socioeconomic Statuses: A Quantitative Meta‐Analysis.” Developmental Science 25, no. 3: e13192. 10.1111/DESC.13192. 34806256

[infa12638-bib-0025] Dargue, N. , N. Sweller , and M. P. Jones . 2019. “When Our Hands Help Us Understand: A Meta‐Analysis into the Effects of Gesture on Comprehension.” Psychological Bulletin 145, no. 8: 765–784. 10.1037/BUL0000202. 31219263

[infa12638-bib-0026] De Klerk, C. C. J. M. , M. L. Filippetti , and S. Rigato . 2021. “The Development of Body Representations: An Associative Learning Account.” Proc Biol Sci 288, no. 1949: 20210070. 10.1098/rspb.2021.0070. 33906399 PMC8079995

[infa12638-bib-0027] de Nooijer, J. A. , T. van Gog , F. Paas , and R. A. Zwaan . 2013. “Effects of Imitating Gestures During Encoding or During Retrieval of Novel Verbs on Children’s Test Performance.” Acta Psychologica 144, no. 1: 173–179. 10.1016/j.actpsy.2013.05.013. 23820099

[infa12638-bib-0028] Dosso, J. A. , and J. P. Boudreau . 2014. “Crawling and Walking Infants Encounter Objects Differently in a Multi‐Target Environment.” Experimental Brain Research 232, no. 10: 3047–3054. 10.1007/s00221-014-3984-z. 24888534

[infa12638-bib-0029] Fenson, L. , P. S. Dale , J. S. Reznick , et al. 1994. “Variability in Early Communicative Development.” Monographs of the Society for Research in Child Development 59, no. 5: i–185. 10.2307/1166093. 7845413

[infa12638-bib-0030] Ferguson, B. , E. Graf , and S. R. Waxman . 2014. “Infants Use Known Verbs to Learn Novel Nouns: Evidence from 15‐ and 19‐Month‐Olds.” Cognition 131, no. 1: 139–146. 10.1016/j.cognition.2013.12.014. 24463934

[infa12638-bib-0031] Franchak, J. M. , K. S. Kretch , and K. E. Adolph . 2018. “See and Be Seen: Infant–Caregiver Social Looking During Locomotor Free Play.” Developmental Science 21, no. 4: 1–13. 10.1111/desc.12626. PMC5920801 29071760

[infa12638-bib-0032] Gampe, A. , J. Brauer , and M. M. Daum . 2016. “Imitation Is Beneficial for Verb Learning in Toddlers.” European Journal of Developmental Psychology 13, no. 5: 594–613. 10.1080/17405629.2016.1139495.

[infa12638-bib-0033] Gentner, D. 1978. “On Relational Meaning: The Acquisition of Verb Meaning.” Child Development 49, no. 4: 988. 10.2307/1128738.

[infa12638-bib-0034] Gentner, D. (1982). Why Nouns Are Learned before Verbs: Linguistic Relativity Versus Natural Partitioning. Technical Report No. 257.

[infa12638-bib-0035] Gentner, D. 2006. “Why Verbs Are Hard to Learn.” In Action Meets Word: How Children Learn Verbs, edited by K. A. Hirsh‐Pasek and R. M. Golinkoff , 544–564. New York: Oxford University Press. 10.1093/acprof:oso/9780195170009.003.0022.

[infa12638-bib-0036] Gentner, D. , and L. Boroditsky . 2001. “Individuation, Relativity, and Early Word Learning.” In Language Acquisition and Conceptual Development, edited by M. Bowerman and S. Levinson , 215–256. Cambridge: Cambridge University Press. 10.1017/cbo9780511620669.010.

[infa12638-bib-0037] Gerson, S. A. , H. Bekkering , and S. Hunnius . 2015. “Short‐term Motor Training, but Not Observational Training, Alters Neurocognitive Mechanisms of Action Processing in Infancy.” Journal of Cognitive Neuroscience 27, no. 6: 1207–1214. 10.1162/jocn_a_00774. 25514654

[infa12638-bib-0038] Gerson, S. A. , and A. L. Woodward . 2012. “A Claw Is like My Hand: Comparison Supports Goal Analysis in Infants.” Cognition 122, no. 2: 181–192. 10.1016/j.cognition.2011.10.014. 22099543 PMC3586806

[infa12638-bib-0039] Gerson, S. A. , and A. L. Woodward . 2014a. “Learning From Their Own Actions: The Unique Effect of Producing Actions on Infants’ Action Understanding.” Child Development 85, no. 1: 264–277. 10.1111/cdev.12115. 23647241 PMC3740060

[infa12638-bib-0040] Gerson, S. A. , and A. L. Woodward . 2014b. “The Joint Role of Trained, Untrained, and Observed Actions at the Origins of Goal Recognition.” Infant Behavior and Development 37, no. 1: 94–104. 10.1016/j.infbeh.2013.12.013. 24468646 PMC3951724

[infa12638-bib-0041] Gillette, J. , H. Gleitman , L. Gleitman , and A. Lederer . 1999. “Human Simulations of Vocabulary Learning.” Cognition 73, no. 2: 135–176. 10.1016/S0010-0277(99)00036-0. 10580161

[infa12638-bib-0042] Gleitman, L. 1990. “The Structural Sources of Verb Meanings.” Language Acquisition 1, no. 1: 3–55. 10.1207/s15327817la0101_2.

[infa12638-bib-0043] Gleitman, L. , and H. Gleitman . 1992. “A Picture Is Worth a Thousand Words, but that’s the Problem: The Role of Syntax in Vocabulary Acquisition.” Current Directions in Psychological Science 1, no. 1: 31–35. 10.1111/1467-8721.ep10767853.

[infa12638-bib-0044] Glenberg, A. M. , and V. Gallese . 2012. “Action‐based Language: A Theory of Language Acquisition, Comprehension, and Production.” Cortex 48, no. 7: 905–922. 10.1016/j.cortex.2011.04.010. 21601842

[infa12638-bib-0045] Goldfield, B. A. , and J. S. Reznick . 1990. “Early Lexical Acquisition: Rate, Content, and the Vocabulary Spurt.” Journal of Child Language 17, no. 1: 171–183. 10.1017/S0305000900013167. 2312640

[infa12638-bib-0046] Goldin‐Meadow, S. , M. E. P. Seligman , and R. Gelman . 1976. “Language in the Two‐Year Old.” Cognition 4, no. 2: 189–202. 10.1016/0010-0277(76)90004-4.

[infa12638-bib-0047] Gonzalez, S. L. , V. Alvarez , and E. L. Nelson . 2019. “Do Gross and Fine Motor Skills Differentially Contribute to Language Outcomes? A Systematic Review.” Frontiers in Psychology 10, no. December: 1–16. 10.3389/fpsyg.2019.02670. 31849775 PMC6901663

[infa12638-bib-0048] Goodman, J. C. , L. McDonough , and N. B. Brown . 1998. “The Role of Semantic Context and Memory in the Acquisition of Novel Nouns.” Child Development 69, no. 5: 1330–1344. 10.1111/j.1467-8624.1998.tb06215.x. 9839419

[infa12638-bib-0049] Hagihara, H. , H. Yamamoto , Y. Moriguchi , and M. aki Sakagami . 2022. “When “Shoe” Becomes Free From “Putting on”: The Link Between Early Meanings of Object Words and Object‐specific Actions.” Cognition 226: 105177. 10.1016/j.cognition.2022.105177. 35653910

[infa12638-bib-0050] Hamilton, A. , K. Plunkett , and G. Schafer . 2000. “Infant Vocabulary Development Assessed With a British Communicative Development Inventory: Lower Scores in the UK Than the USA.” Journal of Child Language 27, no. 3: 689–705. 10.1017/S0305000900004414. 11089344

[infa12638-bib-0051] Harris, P. A. , R. Taylor , B. L. Minor , et al. 2019. “The REDCap Consortium: Building an International Community of Software Platform Partners.” Journal of Biomedical Informatics 95: 103208. 10.1016/j.jbi.2019.103208. 31078660 PMC7254481

[infa12638-bib-0052] Harris, P. A. , R. Taylor , R. Thielke , J. Payne , N. Gonzalez , and J. G. Conde . 2009. “Research Electronic Data Capture (REDCap)‐A Metadata‐Driven Methodology and Workflow Process for Providing Translational Research Informatics Support.” Journal of Biomedical Informatics 42, no. 2: 377–381. 10.1016/j.jbi.2008.08.010. 18929686 PMC2700030

[infa12638-bib-0053] He, M. , E. A. Walle , and J. J. Campos . 2015. “A Cross‐National Investigation of the Relationship Between Infant Walking and Language Development.” Infancy 20, no. 3: 283–305. 10.1111/infa.12071.

[infa12638-bib-0054] Houston‐Price, C. , E. Mather , and E. Sakkalou . 2007. “Discrepancy Between Parental Reports of Infants’ Receptive Vocabulary and Infants’ Behaviour in a Preferential Looking Task.” Journal of Child Language 34, no. 4: 701–724. 10.1017/S0305000907008124. 18062356

[infa12638-bib-0055] Houwen, S. , L. Visser , A. van der Putten , and C. Vlaskamp . 2016. “The Interrelationships Between Motor, Cognitive, and Language Development in Children With and Without Intellectual and Developmental Disabilities.” Research in Developmental Disabilities 53–54: 19–31. 10.1016/j.ridd.2016.01.012. 26851384

[infa12638-bib-0056] Hunnius, S. , and H. Bekkering . 2014. “What Are You Doing? How Active and Observational Experience Shape Infants’ Action Understanding.” Philosophical Transactions of the Royal Society B: Biological Sciences 369, no. 1644: 20130490. 10.1098/rstb.2013.0490. PMC4006192 24778386

[infa12638-bib-0057] Hurt, L. , P. Ashfield‐Watt , J. Townson , et al. 2019. “Cohort Profile: HealthWise Wales. A Research Register and Population Health Data Platform With Linkage to National Health Service Data Sets in Wales.” BMJ Open 9, no. 12: 1–11. 10.1136/bmjopen-2019-031705. PMC7003385 31796481

[infa12638-bib-0058] Huttenlocher, J. , P. Smiley , and R. Charney . 1983. “Emergence of Action Categories in the Child: Evidence From Verb Meanings.” Psychological Review 90, no. 1: 72–93. 10.1037/0033-295X.90.1.72.

[infa12638-bib-0059] Imai, M. , E. Haryu , and H. Okada . 2005. “Mapping Novel Nouns and Verbs onto Dynamic Action Events: Are Verb Meanings Easier to Learn Than Noun Meanings for Japanese Children?” Child Development 76, no. 2: 340–355. 10.1111/j.1467-8624.2005.00849_a.x. 15784086

[infa12638-bib-0060] Imai, M. , E. Haryu , H. Okada , L. Lianjing , and J. Shigematsu . 2006. “Revisiting the Noun‐Verb Debate: A Cross‐Linguistic Comparison of Novel Noun and Verb Learning in English‐Japanese‐and Chinese‐Speaking Children.” Action Meets Word: How Children Learn Verbs: 450–476. 10.1093/ACPROF:OSO/9780195170009.003.0018.

[infa12638-bib-0061] Imai, M. , L. Li , E. Haryu , et al. 2008. “Novel Noun and Verb Learning in Chinese‐English‐and Japanese‐speaking Children.” Child Development 79, no. 4: 979–1000. 10.1111/j.1467-8624.2008.01171.x. 18717902

[infa12638-bib-0062] Iverson, J. M. 2010. “Developing Language in a Developing Body: The Relationship Between Motor Development and Language Development.” Journal of Child Language 37, no. 2: 229–261. 10.1017/S0305000909990432. 20096145 PMC2833284

[infa12638-bib-0063] Iverson, J. M. 2021. “Developmental Variability and Developmental Cascades: Lessons From Motor and Language Development in Infancy.” Current Directions in Psychological Science 30, no. 3: 228–235. 10.1177/0963721421993822. 34194130 PMC8240753

[infa12638-bib-0064] Iverson, J. M. 2022. “Developing Language in a Developing Body, Revisited: The Cascading Effects of Motor Development on the Acquisition of Language.” WIREs Cognitive Science 13, no. 6: e1626. 10.1002/wcs.1626. 36165333 PMC12333486

[infa12638-bib-0065] Jackson‐Maldonado, D. , D. Thal , V. Marchman , E. Bates , and V. Gutierrez‐Clellen . 1993. “Early Lexical Development in Spanish‐speaking Infants and Toddlers.” Journal of Child Language 20, no. 3: 523–549. 10.1017/S0305000900008461. 8300774

[infa12638-bib-0066] Jaeger, T. F. 2008. “Categorical Data Analysis: Away From ANOVAs (Transformation or Not) and Towards Logit Mixed Models.” Journal of Memory and Language 59, no. 4: 434–446. 10.1016/j.jml.2007.11.007. 19884961 PMC2613284

[infa12638-bib-0067] Jones, K. H. , D. V. Ford , C. Jones , et al. 2014. “A Case Study of the Secure Anonymous Information Linkage (SAIL) Gateway: A Privacy‐Protecting Remote Access System for Health‐Related Research and Evaluation.” Journal of Biomedical Informatics 50: 196–204. 10.1016/j.jbi.2014.01.003. 24440148 PMC4139270

[infa12638-bib-0068] Karasik, L. B. , C. S. Tamis‐Lemonda , and K. E. Adolph . 2011. “Transition From Crawling to Walking and Infants’ Actions With Objects and People.” Child Development 82, no. 4: 1199–1209. 10.1111/j.1467-8624.2011.01595.x. 21545581 PMC3163171

[infa12638-bib-0069] Karasik, L. B. , C. S. Tamis‐Lemonda , and K. E. Adolph . 2014. “Crawling and Walking Infants Elicit Different Verbal Responses From Mothers.” Developmental Science 17, no. 3: 388–395. 10.1111/desc.12129. 24314018 PMC3997624

[infa12638-bib-0070] Kassambara, A. 2023. “rstatix: Pipe‐Friendly Framework for Basic Statistical Tests.” R package version 0.7.2. https://rpkgs.datanovia.com/rstatix/.

[infa12638-bib-0071] Kersten, A. W. , and L. B. Smith . 2002. “Attention to Novel Objects During Verb Learning.” Child Development 73, no. 1: 93–109. 10.1111/1467-8624.00394. 14717246

[infa12638-bib-0072] Kim, S. 2015. “ppcor: An R Package for a Fast Calculation to Semi‐partial Correlation Coefficients.” Communications for Statistical Applications and Methods 22, no. 6: 665–674. 10.5351/csam.2015.22.6.665. 26688802 PMC4681537

[infa12638-bib-0073] Kretch, K. S. , J. M. Franchak , and K. E. Adolph . 2014. “Crawling and Walking Infants See the World Differently.” Child Development 85, no. 4: 1503–1518. 10.1111/cdev.12206. 24341362 PMC4059790

[infa12638-bib-0074] Kuchirko, Y. A. , and C. S. Tamis‐LeMonda . 2019. “The Cultural Context of Infant Development: Variability, Specificity, and Universality.” Advances in Child Development and Behavior 57: 27–63. 10.1016/BS.ACDB.2019.04.004. 31296318

[infa12638-bib-0075] Lenth, R. 2024. emmeans: Estimated Marginal Means, Aka Least‐Squares Means. R package version 1.10.2. https://rvlenth.github.io/emmeans/.

[infa12638-bib-0076] Libertus, K. , and R. J. Landa . 2013. “The Early Motor Questionnaire (EMQ): A Parental Report Measure of Early Motor Development.” Infant Behavior and Development 36, no. 4: 833–842. 10.1016/j.infbeh.2013.09.007. 24140841 PMC3858411

[infa12638-bib-0077] Longobardi, E. , P. Spataro , D. L. Putnick , and M. H. Bornstein . 2017. “Do Early Noun and Verb Production Predict Later Verb and Noun Production? Theoretical Implications.” Journal of Child Language 44, no. 2: 480–495. 10.1017/S0305000916000064. 26880050 PMC5822724

[infa12638-bib-0078] Ma, W. , R. M. Golinkoff , K. Hirsh‐Pasek , C. McDonough , and T. Tardif . 2009. “Imageability Predicts the Age of Acquisition of Verbs in Chinese Children.” Journal of Child Language 36, no. 2: 405–423. 10.1017/S0305000908009008. 18937878 PMC2925137

[infa12638-bib-0079] Mani, N. , and F. Huettig . 2012. “Prediction During Language Processing Is a Piece of Cake‐But Only for Skilled Producers.” Journal of Experimental Psychology: Human Perception and Performance 38, no. 4: 843–847. 10.1037/a0029284. 22774799

[infa12638-bib-0080] Maouene, J. , S. Hidaka , and L. B. Smith . 2008. “Body Parts and Early‐Learned Verbs.” Cognitive Science 32, no. 7: 1200–1216. 10.1080/03640210802019997. 21585449

[infa12638-bib-0081] Maouene, J. , N. Sethuraman , A. Laakso , and M. Maouene . 2011. “The Body Region Correlates of Concrete and Abstract Verbs.” Cognition, Brain, Behavior. An Interdisciplinary Journal XV, no. 4: 449–484.

[infa12638-bib-0082] McDonough, C. , L. Song , K. Hirsh‐Pasek , R. M. Golinkoff , and R. Lannon . 2011. “An Image Is Worth a Thousand Words: Why Nouns Tend to Dominate Verbs in Early Word Learning.” Developmental Science 14, no. 2: 181–189. 10.1111/j.1467-7687.2010.00968.x. 21359165 PMC3043374

[infa12638-bib-0083] McQuillan, M. E. , L. B. Smith , C. Yu , and J. E. Bates . 2020. “Parents Influence the Visual Learning Environment through Children’s Manual Actions.” Child Development 91, no. 3: e701–e720. 10.1111/cdev.13274. 31243763 PMC6930973

[infa12638-bib-0084] Moore, C. , S. Dailey , H. Garrison , A. Amatuni , and E. Bergelson . 2019. “Point, Walk, Talk: Links Between Three Early Milestones, From Observation and Parental Report.” Developmental Psychology 55, no. 8: 1579–1593. 10.1037/dev0000738. 31094558 PMC6892347

[infa12638-bib-0085] Muluk, N. B. , B. Bayoğlu , and B. Anlar . 2016. “A Study of Language Development and Affecting Factors in Children Aged 5 to 27 Months.” Ear, Nose & Throat Journal 95, no. 1: 23–29. 10.1177/014556131609500107. 26829690

[infa12638-bib-0086] Naigles, L. 1990. “Children Use Syntax to Learn Verb Meanings.” Journal of Child Language 17, no. 2: 357–374. 10.1017/S0305000900013817. 2380274

[infa12638-bib-0087] Nomikou, I. , M. Koke , and K. J. Rohlfing . 2017. “Verbs in Mothers’ Input to Six‐Month‐Olds: Synchrony Between Presentation, Meaning, and Actions Is Related to Later Verb Acquisition.” Brain Sciences 7, no. 5: 52. 10.3390/brainsci7050052. 28468265 PMC5447934

[infa12638-bib-0088] Oakes, L. M. , and D. H. Rakison . 2020. Developmental Cascades: Building the Infant Mind. New York: Oxford University Press.

[infa12638-bib-0089] Oudgenoeg‐Paz, O. , M. C. J. M. Volman , and P. P. M. Leseman . 2012. “Attainment of Sitting and Walking Predicts Development of Productive Vocabulary Between Ages 16 and 28 Months.” Infant Behavior and Development 35, no. 4: 733–736. 10.1016/j.infbeh.2012.07.010. 22982273

[infa12638-bib-0090] Pereira, A. F. , L. B. Smith , and C. Yu . 2014. “A Bottom‐Up View of Toddler Word Learning.” Psychonomic Bulletin & Review 21, no. 1: 178–185. 10.3758/s13423-013-0466-4. 23813190 PMC3883952

[infa12638-bib-0091] Piaget, J. 1952. The Origins of Intelligence in Children. New York: International Universities Press.

[infa12638-bib-0092] Pomiechowska, B. , G. Bródy , G. Csibra , and T. Gliga . 2021. “Twelve‐month‐olds Disambiguate New Words Using Mutual‐Exclusivity Inferences.” Cognition 213: 104691. 10.1016/j.cognition.2021.104691. 33934847

[infa12638-bib-0093] Pomiechowska, B. , and T. Gliga . 2019. “Lexical Acquisition through Category Matching: 12‐Month‐Old Infants Associate Words to Visual Categories.” Psychological Science 30, no. 2: 288–299. 10.1177/0956797618817506. 30575444

[infa12638-bib-0094] Rescorla, L. , Y. M. Cathy Lee , K. J. Oh , and Y. A. Kim . 2013. “Lexical Development in Korean: Vocabulary Size, Lexical Composition, and Late Talking.” Journal of Speech, Language, and Hearing Research 56, no. 2: 735–747. 10.1044/1092-4388(2012/11-0329. 23275411

[infa12638-bib-0095] Rochat, P. 1989. “Object Manipulation and Exploration in 2‐ to 5‐Month‐Old Infants.” Developmental Psychology 25, no. 6: 871–884. 10.1037/0012-1649.25.6.871.

[infa12638-bib-0096] Rochat, P. , and N. Goubet . 1995. “Development of Sitting and Reaching in 5‐ to 6‐Month‐Old Infants.” Infant Behavior and Development 18, no. 1: 53–68. 10.1016/0163-6383(95)90007-1.

[infa12638-bib-0097] Ruddy, M. G. , and M. H. Bornstein . 1982. “Cognitive Correlates of Infant Attention and Maternal Stimulation over the First Year of Life.” Child Development 53, no. 1: 183. 10.2307/1129651. 7060421

[infa12638-bib-0098] Ruff, H. A. 1984. “Infants’ Manipulative Exploration of Objects: Effects of Age and Object Characteristics.” Developmental Psychology 20, no. 1: 9–20. 10.1037/0012-1649.20.1.9.

[infa12638-bib-0099] Samuelson, L. K. , and L. B. Smith . 1998. “Memory and Attention Make Smart Word Learning: An Alternative Account of Akhtar, Carpenter, and Tomasello.” Child Development 69, no. 1: 94. 10.2307/1132073. 9499560

[infa12638-bib-0100] Schneider, J. L. , and J. M. Iverson . 2022. “Cascades in Action: How the Transition to Walking Shapes Caregiver Communication During Everyday Interactions.” Developmental Psychology 58, no. 1: 1–16. 10.1037/dev0001280. 34843275 PMC9588170

[infa12638-bib-0101] Schroer, S. E. , and C. Yu . 2022. “Looking Is Not Enough: Multimodal Attention Supports the Real‐Time Learning of New Words.” Developmental Science 26, no. 2: e13290. 10.1111/DESC.13290. 35617054

[infa12638-bib-0102] Sharon, T. , and K. Wynn . 1998. “Individuation of Actions From Continuous Motion.” Psychological Science 9, no. 5: 357–362. 10.1111/1467-9280.00068.

[infa12638-bib-0103] Smith, L. B. , S. S. Jones , and B. Landau . 1996. “Naming in Young Children: A Dumb Attentional Mechanism?” Cognition 60, no. 2: 143–171. 10.1016/0010-0277(96)00709-3. 8811743

[infa12638-bib-0104] Smith, L. B. , and C. Yu . 2008. “Infants Rapidly Learn Word‐Referent Mappings via Cross‐Situational Statistics.” Cognition 106, no. 3: 1558–1568. 10.1016/j.cognition.2007.06.010. 17692305 PMC2271000

[infa12638-bib-0105] Snedeker, J. , and L. Gleitman . 2004. “Why It Is Hard to Label Our Concepts.” In Weaving a Lexicon, edited by D. G. Hall and S. R. Waxman , 257–293. Cambridge, MA: MIT Press.

[infa12638-bib-0106] Sootsman Buresh, J. , A. Woodward , and C. W. Brune . 2006. “The Roots of Verbs in Prelinguistic Action Knowledge.” In Action Meets Word: How Children Learn Verbs, edited by K. Hirsh‐Pasek and R. M. Golinkoff . New York: Oxford University Press. 10.1093/ACPROF:OSO/9780195170009.003.0009.

[infa12638-bib-0107] Soska, K. C. , and K. E. Adolph . 2014. “Postural Position Constrains Multimodal Object Exploration in Infants.” Infancy 19, no. 2: 138–161. 10.1111/infa.12039. 24639621 PMC3951720

[infa12638-bib-0108] Spelke, E. S. , R. Kestenbaum , D. J. Simons , and D. Wein . 1995. “Spatiotemporal Continuity, Smoothness of Motion and Object Identity in Infancy.” British Journal of Developmental Psychology 13, no. 2: 113–142. 10.1111/j.2044-835X.1995.tb00669.x.

[infa12638-bib-0109] Styles, S. , and K. Plunkett . 2009. “What Is Word Understanding for the Parent of a One‐Year‐Old? Matching the Difficulty of a Lexical Comprehension Task to Parental CDI Report.” Journal of Child Language 36, no. 4: 895–908. 10.1017/S0305000908009264. 19079845

[infa12638-bib-0110] Suarez‐Rivera, C. , E. Linn , and C. S. Tamis‐LeMonda . 2022. “From Play to Language: Infants’ Actions on Objects Cascade to Word Learning.” Language Learning 72, no. 4: 1092–1127. 10.1111/LANG.12512.

[infa12638-bib-0111] Tacke, N. F. , L. S. Bailey , and M. W. Clearfield . 2015. “Socio‐economic Status (SES) Affects Infants’ Selective Exploration.” Infant and Child Development 24, no. 6: 571–586. 10.1002/icd.1900.

[infa12638-bib-0112] Tamis‐LeMonda, C. S. , S. Custode , Y. Kuchirko , K. Escobar , and T. Lo . 2019. “Routine Language: Speech Directed to Infants During Home Activities.” Child Development 90, no. 6: 2135–2152. 10.1111/CDEV.13089. 29766498

[infa12638-bib-0113] Tamis‐Lemonda, C. S. , Y. Kuchirko , and L. Tafuro . 2013. “From Action to Interaction: Infant Object Exploration and Mothers’ Contingent Responsiveness.” IEEE Transactions on Autonomous Mental Development 5, no. 3: 202–209. 10.1109/TAMD.2013.2269905.

[infa12638-bib-0114] Tamis‐Lemonda, C. S. , L. Song , A. S. Leavell , R. Kahana‐Kalman , and H. Yoshikawa . 2012. “Ethnic Differences in Mother–Infant Language and Gestural Communications Are Associated With Specific Skills in Infants.” Developmental Science 15, no. 3: 384–397. 10.1111/J.1467-7687.2012.01136.X. 22490178

[infa12638-bib-0115] Tardif, T. 1996. “Nouns Are Not Always Learned Before Verbs: Evidence From Mandarin Speakers’ Early Vocabularies.” Developmental Psychology 32, no. 3: 492–504. 10.1037/0012-1649.32.3.492.

[infa12638-bib-0116] Tardif, T. , S. A. Gelman , and F. Xu . 1999. “Putting the “Noun Bias” in Context: A Comparison of English and Mandarin.” Child Development 70, no. 3: 620–635. 10.1111/1467-8624.00045.

[infa12638-bib-0117] Thelen, E. 2000a. “Grounded in the World: Developmental Origins of the Embodied Mind.” Infancy 1, no. 1: 3–28. 10.1207/S15327078IN0101_02. 32680313

[infa12638-bib-0118] Thelen, E. 2000b. “Motor Development as Foundation and Future of Developmental Psychology.” International Journal of Behavioral Development 24, no. 4: 385–397. 10.1080/016502500750037937.

[infa12638-bib-0119] Thelen, E. , and L. B. Smith . 1996. A Dynamic Systems Approach to the Development of Cognition and Action. Cambridge, MA: MIT Press.

[infa12638-bib-0120] Tincoff, R. , and P. W. Jusczyk . 1999. “Some Beginnings of Word Comprehension.” Psychological Science 10, no. 2: 172–175. 10.1111/1467-9280.00127.

[infa12638-bib-0121] Tincoff, R. , and P. W. Jusczyk . 2012. “Six‐Month‐Olds Comprehend Words That Refer to Parts of the Body.” Infancy 17, no. 4: 432–444. 10.1111/j.1532-7078.2011.00084.x. 32693484

[infa12638-bib-0122] Tomasello, M. 1992a. First Verbs: A Case Study of Early Grammatical Development. Cambridge: Cambridge University Press.

[infa12638-bib-0123] Tomasello, M. 1992b. “The Social Bases of Language Acquisition.” Social Development 1, no. 1: 67–87. 10.1111/j.1467-9507.1992.tb00135.x.

[infa12638-bib-0124] Tomasello, M. 1995. “Pragmatic Contexts for Early Verb Learning.” In Beyond Names for Things: Young Children’s Acquisition of Verbs, edited by M. Tomasello and W. E. Merriman , 115–146. New York: Psychology Press.

[infa12638-bib-0125] Tomasello, M. , and C. B. Mervis . 1994. “The Instrument Is Great, but Measuring Comprehension Is Still a Problem.” Monographs of the Society for Research in Child Development 59, no. 5: 174–179. 10.1111/j.1540-5834.1994.tb00186.x.

[infa12638-bib-0126] Townson, J. , J. Davies , L. Hurt , P. Ashfield‐Watt , and S. Paranjothy . 2020. “Developing and Evaluating a Model of Public Involvement and Engagement Embedded in a National Longitudinal Study: HealthWise Wales.” International Journal of Population Data Science 5, no. 3: 1356. 10.23889/IJPDS.V5I3.1356. 34007884 PMC8110888

[infa12638-bib-0127] Valla, L. , K. Slinning , R. Kalleson , T. Wentzel‐Larsen , and K. Riiser . 2020. “Motor Skills and Later Communication Development in Early Childhood: Results from a Population‐Based Study.” Child: Care, Health and Development 46, no. 4: 407–413. 10.1111/cch.12765. 32191337

[infa12638-bib-0128] Vong, W. K. , W. Wang , A. E. Orhan , and B. M. Lake . 2024. “Grounded Language Acquisition through the Eyes and Ears of a Single Child.” Science 383, no. 6682: 504–511. 10.1126/science.adi1374. 38300999

[infa12638-bib-0129] Walle, E. A. , and J. J. Campos . 2014. “Infant Language Development Is Related to the Acquisition of Walking.” Developmental Psychology 50, no. 2: 336–348. 10.1037/a0033238. 23750505

[infa12638-bib-0130] West, K. L. , K. K. Fletcher , K. E. Adolph , and C. S. Tamis‐LeMonda . 2022. “Mothers Talk About Infants’ Actions: How Verbs Correspond to Infants’ Real‐Time Behavior.” Developmental Psychology 58, no. 3: 405–416. 10.1037/dev0001285. 35286106 PMC9012493

[infa12638-bib-0131] West, K. L. , and J. M. Iverson . 2017. “Language Learning Is Hands‐On: Exploring Links Between Infants’ Object Manipulation and Verbal Input.” Cognitive Development 43, no. January 2016: 190–200. 10.1016/j.cogdev.2017.05.004.

[infa12638-bib-0132] West, K. L. , and J. M. Iverson . 2021. “Communication Changes when Infants Begin to Walk.” Developmental Science 24, no. 5: 1–57. 10.1111/desc.13102. PMC8349384 33556219

[infa12638-bib-0133] West, K. L. , A. N. Saleh , K. E. Adolph , and C. S. Tamis‐LeMonda . 2023. ““Go, Go, Go!” Mothers’ Verbs Align With Infants’ Locomotion.” Developmental Science 26, no. 6: e13397. 10.1111/desc.13397. 37078147 PMC10653669

[infa12638-bib-0134] Wynn, K. 1996. “Infants’ Individuation and Enumeration of Actions.” Psychological Science 7, no. 3: 164–169. 10.1111/j.1467-9280.1996.tb00350.x.

[infa12638-bib-0135] Yoshida, H. , and L. B. Smith . 2008. “What’s in View for Toddlers? Using a Head Camera to Study Visual Experience.” Infancy 13, no. 3: 229–248. 10.1080/15250000802004437. 20585411 PMC2888512

[infa12638-bib-0136] Yu, C. , and L. B. Smith . 2007. “Rapid Word Learning under Uncertainty via Cross‐Situational Statistics.” Psychological Science 18, no. 5: 414–420. 10.1111/j.1467-9280.2007.01915.x. 17576281

[infa12638-bib-0137] Yu, C. , and L. B. Smith . 2012. “Embodied Attention and Word Learning by Toddlers.” Cognition 125, no. 2: 244–262. 10.1016/j.cognition.2012.06.016. 22878116 PMC3829203

[infa12638-bib-0138] Zuccarini, M. , A. Guarini , J. M. Iverson , et al. 2018. “Does Early Object Exploration Support Gesture and Language Development in Extremely Preterm Infants and Full‐Term Infants?” Journal of Communication Disorders 76, no. March: 91–100. 10.1016/j.jcomdis.2018.09.004. 30300842

