# Supplementary material for: Parent‐Reported Relations Between Vocabulary and Motor Development in Infancy: Differences Between Verbs and Nouns
Source: Infancy. 2024 Nov 21;30(1):e12638. doi: 10.1111/infa.12638 (PMC11582352; doi:10.1111/infa.12638)
Supplement: Supplementary file 1 — Supplementary Material [file INFA-30-0-s001.docx]

# Parent-Reported Relations Between Vocabulary and Motor Development in Infancy: Differences Between Verbs and Nouns

Supplementary Materials

This document contains mixed effect model comparisons for data fit and results from additional analyses. The data and analysis scripts can be found at <https://osf.io/zwy5k/?view_only=17ea51cb31b84c41bdfb1d00b5343da4>

1. To establish the random structure of our primary analysis (i.e., logistic mixed effect model exploring the association between motor skills and word comprehension for verbs and nouns) three models were established; (a) maximal random structure (described in the main text), (b) a random intercept only model, and (c) a simple random slope model. The model structure of (b) and (c) were as follows:

(b) Word Comprehension ~ EMQ Score* Word Type + Age + (1 | Infants) + (1 | Word Items)

(c) Word Comprehension ~ EMQ Score* Word Type + Age + (1 + Word Type | Infants) + (1 | Word Items)

Following estimation, each model containing random slopes was compared against the random intercept only model using a Chi Square difference test χ^2^. Both models significantly improved the model fit. As such, the model with the lowest AIC (Akaike Information Criterion), a descriptive parameter of model fit, was selected (i.e., maximal random effect structure). See the following table for details.

| Model | AIC | BIC | Loglik | Deviance | χ^2^ | df | *p*-value |
| --- | --- | --- | --- | --- | --- | --- | --- |
| Random Intercepts | 26254 | 26314 | -13120 | 26240 |  |  |  |
| One Random Slope | 25927 | 26004 | -12954 | 25909 | 331.38 | 2 | <.001 |
| Maximal Structure | 25779 | 25899 | -12876 | 25751 | 489.09 | 7 | <.001 |

2. We also repeated the main logistic mixed effects model analysis with parent education and infant sex included as control variables. We used the following *lme4* structure:

Word Comprehension ~ EMQ Score* Word Type + Infant Age + Parent Education + Infant Sex + (1 + Word Type | Infants) + (1 + EMQ Score + Infant Age | Word Items)

The analysis revealed the same pattern of results – parent education and infant sex were not significant. The analysis revealed the same pattern of results across fixed effects and interactions. Specifically, the model revealed a significant interaction between motor skills and word type (*B* = -0.38, CI 95% [-0.65,-0.10], *SE* = 0.14, *p* =. 008), with the association between motor skills and word comprehension significantly greater for verbs than for nouns. The analysis also revealed a significant main effect of motor skills with greater motor ability being associated with increased likelihood of comprehending a word (*B* = 1.71, CI 95% [0.90, 2.51], *SE* = 0.41, *z* = 4.15, *p* <. 001). Again, the model revealed a significant main effect of word type (*B* = -0.91, CI 95% [-1.47, -0.36], *SE* = 0.28, *z* = -3.21, *p* =. 001) with the infant of average age and EMQ score understanding a great proportion of verbs (53.4%) than nouns (43.7%). Age was not a significant fixed effect (*B* = 0.78, CI 95% [-0.01, 1.58], *SE* = 0.40, *z* = 1.94, *p* =. 053). Additional control variables of infant sex and parent education were also not significant (*B* = -0.50, CI 95% [-1.16, 0.17], *SE* = 0.34, *z* = -1.46, *p* = .143; *B* = 0.01, CI 95% [-0.25, 0.27], *SE* = 0.13, *z* = 0.1, *p* = .923), respectively.

3. We also repeated the logistic mixed effects model analysis conducted to assess our primary research question with *only* the original O-CDI verb items included to ascertain whether including additional verbs, that may be comprehended earlier in infancy, biased the results. This resulted in 1411 responses (i.e., 17 verbs per infant) being removed from the analysis. We applied the same random effects structure described in the main text:

Word Comprehension ~ EMQ Score* Word Type + Infant Age + (1 + Word Type | Infants) + (1 + EMQ Score + Infant Age | Word Items)

The analysis revealed the same pattern of results across fixed effects and interactions. Specifically, the model revealed a significant interaction between motor skills and word type (*B* = -0.35, CI 95% [-0.63,-0.07], *SE* = 0.14, *z* = -2.42, *p* =. 015), with the association between motor skills and word comprehension significantly greater for verbs than for nouns (*estimate* = 0.437, CI 95% [0.084, 0.791], *SE* = 0.180, *z* = 2.42, *p* =. 015). The analysis also revealed a significant main effect of motor skills with greater motor ability being associated with increased likelihood of comprehending a word (*B* = 1.74, CI 95% [0.93,2.56], *SE* = 0.42, *z* = 4.18, *p* <. 001). Again, the model revealed a significant main effect of word type (*B* = -0.99, CI 95% [-1.59,-0.38], *SE* = 0.31, *z* = -3.21, *p* =. 001) with the infant of average age and EMQ score understanding a great proportion of verbs (53.4%) than nouns (43.7%). Age was not a significant fixed effect (*B* = 0.71, CI 95% [-0.09,1.50], *SE* = 0.41, *z* = 1.74, *p* =. 081).
